# On the determinants and the role of the payers in the uptake of genetic testing and data sharing in personalized health

**DOI:** 10.3389/fpubh.2023.920286

**Published:** 2023-03-02

**Authors:** Veronika Kalouguina, Joël Wagner

**Affiliations:** ^1^Department of Actuarial Science, Faculty of Business and Economics, University of Lausanne, Lausanne, Switzerland; ^2^Swiss Finance Institute, University of Lausanne, Lausanne, Switzerland

**Keywords:** personalized health, genetic testing, willingness to test, data sharing, preferences, survey study

## Abstract

**Background:**

New health technologies and data offer tailored prevention and spot-on treatments, which can considerably reduce healthcare costs. In healthy individuals, insurers can participate in the creation of health capital through data and preventing the occurrence of a disease. In the onset of a disease, sequencing an individual's genome can provide information leading to the use of more efficient treatments. Both improvements are at the core of the “personalized health” paradigm. As a positive side effect, a reduction in healthcare costs is expected. However, the integration of personalized health in insurance schemes starts with a closer understanding of the demand drivers.

**Methods:**

Using novel data from a survey carried out in Switzerland, we determine the factors influencing the uptake and sharing of data from genetic tests. In our regression analyses, we use five sets of socioeconomic, lifestyle, health insurance, sentiment, and political beliefs variables. Furthermore, two framings assess the willingness to undertake a test and the readiness to share results with an insurer when the costs of the test are borne by the insurer or the individual.

**Results:**

We find that socioeconomic, lifestyle, or political belief variables have very little influence on the uptake of tests and the sharing of data. On the contrary, our results indicate that sentiment and insurance factors play a strong role. More precisely, if genetic tests are perceived as a mean to perform health prevention, this pushes individuals to take them. Furthermore, using the insurer's smartphone app leads to an increase of the likelihood to undergo a test and doubles the probability to share related data. Regarding insurance plans and deductible levels, there is no strong correlation neither with the willingness to take a test nor to share the data. Finally, individuals with complementary health insurance plans are less likely to share results. From the framings for the payment of genetic tests, our results indicate a positive effect of the insurer as a payer on the willingness to undertake tests as well as on data sharing.

**Conclusion:**

Our results lay the ground for a deeper understanding of the role of payers on health decisions and sharing of health-related data. In particular, we find that it is relevant for health insurers to engage with their clients.

## 1. Introduction

Genetic tests (GTs) have several purposes: in the case of a healthy individual, sequencing parts of the genome helps to evaluate the risk of developing a certain disease as well as to pass it to the next generation (1). Newborn screening can reveal disorders that need early medication. Diagnose testing, which happens in the case of a sick individual, allows the medical team to understand the genetic root of the condition and to select the treatment minimizing adverse drug events (2, 3). Finally, direct-to-consumer GTs allow individuals to obtain a genetic screening without healthcare intermediaries. The tests can provide genetic-based food intolerances, exercise plans, and, in certain cases, a risk profile for specific diseases such as breast cancer [see, e.g., Su (4)].^1^

GTs rely on very strong power of data. Translating genetic information gives the individual knowledge about the own risk level of a disease and hence the leverage to act on it. For example, by changing the lifestyle and health behaviors (9), one can reduce the probability of the occurrence of a disease. Furthermore, the results of the GT enable enlightened decisions to schedule a personalized check-up plan for the individual and to monitor those specific risks (10). Finally, researchers as actors of the health ecosystem can run analyses with the anonymized data to understand which types of prevention work best for which predisposition to a disease. From a social sciences perspective, among the first steps to unlock the benefits of GTs, is to understand what drives individuals to take them. To grasp the general public attitude toward GTs and its willingness to undergo a GT (“genetic testing willingness,” GTW) provides policymakers and stakeholders (e.g., insurers) with insights useful to promote their uptake. However, so far, several authors in the literature solely focus on a particular condition to assess GTW. For instance, cancer susceptibility risk assessment is a recurrent subject under study [see, e.g., Fogel et al. (11)]. Often, the surveyed population and the criteria for an admittance in a particular study usually include family history and being at risk for a given disease (12).

In this article, to fill the gap, we provide a general study of the GTW and the willingness to share the related data (“data sharing willingness,” DSW), using novel data from an *ad hoc* survey carried out in Switzerland. We determine the factors influencing the uptake and sharing of data from GTs. Through regression analyses followed by a random forest robustness check, we use five sets of variables, socioeconomic, lifestyle, health insurance, sentiment, and political beliefs, and two framings. The two framings assess the GTW and the DSW of anonymized test results with the health insurer when the costs of the tests are either borne by the insurer or by the individual. Moreover, our survey design adds the effect of the payer dimension to our analysis through the framings. Including the health insurer as an actor has seldom been done and brings new results to this pane of the literature.

Our article hence focuses on two research questions:

1. What factors explain the GTW? To assess this question, we consider regressions with five categories of variables, namely, socioeconomic, lifestyle, insurance, political, and sentiment factors. We first regress them by categories separately and in a total regression subsequently. To test the robustness of our results, we use a random forest approach on the total regression model to get an importance ranking of the effects.
2. What role does the payer play in the GTW and DSW? To answer our second research question, we have designed a survey which not only solely allows for the inclusion of various factors but also captures the effect of the insurer as a payer in an additional manner by framing. The framing consists of dividing the sample into two equally sized subsamples, with each sample being presented with a different framing. After presenting the price range for GTs, we display two different sentences introducing two different payers. For the first subsample, the framing suggests that the GTs should be paid by the health insurer. For the second group, it says that the individual him/herself should pay for the test.

We find that socioeconomic, lifestyle, or political belief variables have very little or no influence on the uptake of GTs and the sharing of the results with an insurer, which is in line with the literature (13). On the contrary, our results indicate that insurance and sentiment factors play a strong role. More precisely, if GTs are perceived as a mean to perform health prevention, this pushes individuals to take them by an increase in propensity of 10.9 pp. Furthermore, using the health insurer's smartphone app leads to an increase of 16.5% in the GTW and of 27.6% to anonymously share the related data with the insurer. Regarding insurance plans and deductible levels, there is no strong correlation neither with the GTW nor with the DSW. Finally, individuals with complementary health insurance plans are less likely to share anonymized test results with their insurer. Using framings for the payment of GTs, we seize the effect of the insurer as a payer on both GTW and DSW. Our results indicate a positive effect of the insurer as a payer on the GTW (+24.8%) as well as the DSW with the health insurer (+9.4%).

The remaining of the paper is organized as follows: Section 2 offers a literature review along the research questions and the methodology, as well as a description of the variables with descriptive statistics. Section 3 present both regression and random forest results. Finally, in Section 4, we conclude and provide a discussion for further research.

## 2. Materials and methods: Literature review, survey setup, and descriptive statistics

### 2.1. Literature review

The state of the existent literature is described by Sweeny et al. (13) as being “[...] rife with conflicting findings, inconsistent methodology, and uneven attention across test types and across predictors of genetic testing decisions.” One can find several clusters of studies in the academic research. They differ either by the nature of the GTs submitted for questioning or by the population under study. First, extant research mostly focuses on the GTW or the willingness to pay for a particular GT related to a certain disease, such as breast cancer [e.g., Armstrong et al. (14)], Alzheimer's disease [e.g., Kopits et al. (15)], or colon cancer [e.g., Lerman et al. (16)]. Few articles query on the willingness to do or to pay for GTs in general. Second, in many studies, the subject population is targeted and not randomly selected. The selection of the sample is usually based on criteria such as being at-risk for a certain condition. For instance, in the case of Dalpe et al. (12), women between 35 and 55 years were inquired about their interest to undergo GTs in search of a mutation which may lead to breast cancer. Following this restricted selection, the sample size usually ends up in < 1,000 individuals. Finally, a lot of research is conducted under a social sciences perspective rather than a economical view point; hence, we found very seldom health insurance as being an examined factor for the GTW. When the health insurer was mentioned, it was mostly presented in the perceived barriers section as a possible discriminator following a GT. As an instance, the fear of denial for coverage is discussed in multiple articles [e.g., Hall et al. (17), Allain et al. (18), Haga et al. (19), and Clayton et al. (20)].

Regarding drivers of the decision to take a GT, socioeconomic factors are often assessed. They include age, gender, education, employment status, marital status, and income. Throughout our literature review, we did not find consistent results for any of these factors. For instance, in Armstrong et al. (14) and Miron-Shatz et al. (21), older women are more likely to undergo genetic testing for breast cancer than younger ones. In Tubeuf et al. (22) or Wessel et al. (23), however, age does not play a role in the interest for genetic testing for retinal disease or diabetes type 2, respectively. These conflicting findings are backed up by Sweeny et al. (13), in their literature review. The authors find likewise that age has an unclear outcome on decision-making for genetic testing. They also have assessed the effects of the aforementioned socioeconomic factors and the results are the same to what is observed more recently by Wessel et al. (23). Regarding socioeconomic factors, the results found in the literature do not reach a consensus either. Predictors such as gender, education, income, or marital status present different effects on the decisions-taking. Throughout the articles, results are ranging from a positive to negative effect with most studies not giving conclusive results.

Another interesting factor is the family health history, i.e., the existence or not in the close family of an individual who is suffering or suffered from a given health condition. Expectedly, in a majority of articles, the existence of a family member bearing a particular condition leads to an increase in the likelihood of the individual to undergo genetic testing. Blouin-Bougie et al. (24), Abdul Rahim et al. (25), and Sun et al. (26), to cite a few, document such results. Interestingly, a research on a sample of 1,960 British individuals by Sanderson et al. (27) presented opposing findings for the GTW for heart disease or cancer predisposition. In their results, individuals with a family history of heart disease are more likely to do a GT for heart disease, whereas individuals with cancer running in their family are less likely to undergo a GT for cancer. Again, in their systematic review of the literature, Sweeny et al. (13) confirmed that family health history displays either a positive relationship with GTs or no statistical relevance.

Despite the heterogeneity in socioeconomic factors, the literature nevertheless presents several consistent drivers displaying a clear effect on the willing to do or to pay. These drivers are psychological and they reflect the individual's view of the gains or losses a GT may result in. They are usually part of the health belief model (28), more precisely the perceived benefits and barriers of the tests, health motivations, and perceived susceptibility or severity. The most extensive literature is found on the effect of perceived benefits of genetic testing. These benefits can take several forms like the knowledge about the risks of getting a particular condition [e.g., Gollust et al. (29), Wessel et al. (23), Fogel et al. (11), Kauffman et al. (30), and Abdul Rahim et al. (25)], have adequate prevention [e.g., Lerman et al. (16) and Alanazy et al. (31)], or inform relatives of a possible risk [e.g., Smith and Croyle (32), Armstrong et al. (14), Hall et al. (17), Fogel et al. (11), and Sun et al. (26)]. These benefits are incentives for individuals to undergo testing and hence have a positive impact on the GTW. This is consistent and statistically significant throughout the literature (13). The perceived barriers also play a role in the GT uptake decision. The most common fears are the financial consequences of the testing [e.g., Bosompra et al. (33), Alanazy et al. (31), and Sun et al. (26)] and the possible discrimination by employers and insurers [e.g., Lerman et al. (16), Armstrong et al. (14), Cameron et al. (34), and Dalpe et al. (12)].

In regard of the literature gaps and research avenues presented earlier, the aim of our research is two-fold. Our study builds on an original survey to address several gaps in the literature. By randomly selecting a representative sample of participants, we ensure the understanding of the GTW and of the DSW in a broad, lay population. In addition, the size of the sample gives us the opportunity to add a dimension using the payer of the GT as a framing.

### 2.2. Survey setup

To conduct our study, we created an original survey for which the collection of data was supported by a polling agency. The sample comprises 1,000 respondents from Switzerland evenly distributed by gender, by four age categories between 25 and 65 years, and by language regions with two-thirds from the German-speaking part and one-third from the French-speaking part.

After briefly explaining the purpose of GTs in the context of personalized health, we inquire individuals whether they are using or would be willing to use such type of technology. Subsequently, we focus on GTs and question individuals about factors which could incentivize or refrain them from performing such a test. We take advantage of this focused section to also analyze the effect of the price and the payer on individual's enthusiasm to do the GT through framing with different scenarios. Finally, we ask socioeconomic, sentiment, and political questions.

#### 2.2.1. Response variables: Would you carry out such a genetic test?

The core of our questionnaire starts with an introductory paragraph providing the basic knowledge for the surveyed individuals and to set boundaries for a common understanding of genetic testing in the present research.

**Figure 1:**
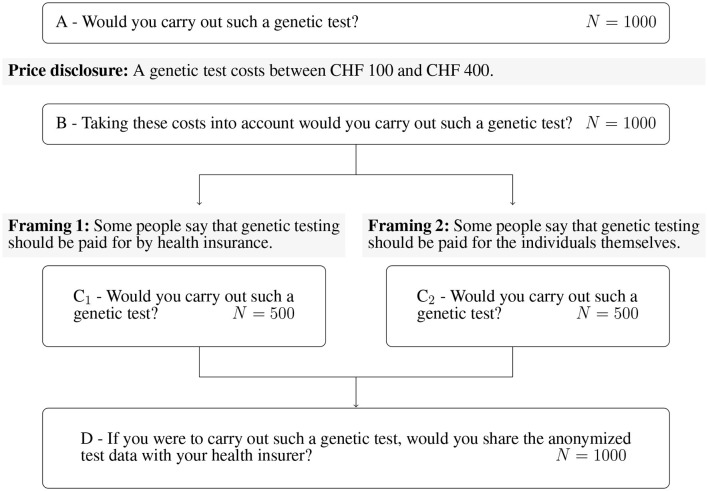
Survey setup, core questions, and framings.

In Figure 1, one can observe that we first question the GTW without price information (questions A and B). Subsequently, the whole sample is then divided into two subsamples of randomly selected 500 individuals. The framing targets the payer of the GT. In Framing 1, the payer is the health insurer (question C1), whereas in Framing 2, it is the individual (question C2). Once the question about GTW following the framing is asked, both subsamples are inquired about the DSW of anonymized data with the health insurer (question D). We report relevant excerpts of the questionnaire in the Supplementary material. The questions A, B, C1, C2, and D correspond to the questions C3, C4, C5c, C5d, and C6, respectively.

#### 2.2.2. Explanatory variables

The first questions of our survey selected the participants. These questions inquired about age, gender, and postal code to select the respondents, and balanced the panel according to the criteria. The majority of the other questions leading to our explanatory variables were asked after the core questions. The first set of questions relates to socioeconomic factors and is composed of 10 variables. The second and third sets are insurance and lifestyle factors, containing four and six variables, respectively. The fourth set is made of political factors with three variables. The last set is the largest, assembling 24 variables regarding sentiment factors. Several variables present binary categories. Indeed, for some of them, the original categories were merged to create a binary outcome as to decrease the length of the model and avoid a potential overfit. Table 1 provides the list of all the used variables and a brief description of the variable itself, accompanied by the available categories, along the five sets of variables.

**Table 1:** Summary of the variables along the five sets.

**Variable**	**Description**	**Categories**
**Socioeconomic factors**		
Gender	Gender of the respondent	Male, female
Age	Age class in years	25–34, 35–44, 45–54, 55–65
Region of residency	Canton defined by the spoken language	French-speaking, German-speaking
Nationality	Nationality of the respondent	Other, Swiss
Education	Higher education (above high school level)	No, yes
Professional situation	Current employment situation	Full-time employed, part-time employed, other
Subjective wealth	Subjective household wealth	Below average, above average
Marital status	Marital status	Married/registered partnership, other
Health	Self-rated health	Bad, average, good
Cancer history	History of cancer, cardiac or hereditary disease in close family	No, yes
**Lifestyle factors**		
Alcohol consumption	Alcohol consumption	Everyday, sometimes, never
Cigarette consumption	Smoking habit	Everyday, sometimes, never
Greens consumption	Fruits and vegetables consumption	Everyday, sometimes, never
Sport	Exercising habit	At least once a week, less
Future planning	Interest of planing for the future	0 to 1 by increments of 0.1
Risk-loving	Readiness to take risks	0 to 1 by increments of 0.1
**Insurance factors**		
Insurance plan	Mandatory health insurance plan	Basic, Health Maintenance Organization, family doctor, CallMed
Deductible	Mandatory health insurance level of deductible	CHF 300, 500–2,000, 2,500
Complementary insurance	Complementary health insurance	No, yes
Insurer's app	Insurer's app for step or exercise count	No, yes
**Political factors**		
Interest in politics	Interest in politics	No, yes
Political orientation	Political orientation assigned on the left	0 to 1 by increments of 0.1
Feeling close to a political party	Feeling close to a political party	No, yes
**Sentiment factors**		
Incentive: curiosity	Curiosity is an incentive to undergo genetic testing	No, yes
Incentive: better health prevention	Take better care of health is an incentive to undergo genetic testing	No, yes
Incentive: help relatives prevention	Help relatives to take better care of their health is an incentive to undergo genetic testing	No, yes
Incentive: incentivize relatives	Incentivize relatives is an incentive to undergo genetic testing	No, yes
Incentive: disease risk information	Disease risk information is an incentive to undergo genetic testing	No, yes
Barrier: fear of discrimination	Fear of discrimination is a barrier to undergo genetic testing	No, yes
Barrier: test too costly	Fear of cost of test is a barrier to undergo genetic testing	No, yes
Barrier: family disapproves	Fear of family disapproving is a barrier to undergo genetic testing	No, yes
Barrier: induced lifestyle changes	Induced lifestyle changes is a barrier to undergo genetic testing	No, yes
Barrier: not want info	Not wanting to know the risks is a barrier to undergo genetic testing	No, yes
Barrier: family finances	Impact on family finances is a barrier to undergo genetic testing	No, yes
Impact: more difficult family insured	It will be more difficult for my family to get insured	No, yes
Impact: longer and better life	Genetic testing will promote a healthier and longer life	No, yes
Impact: testing will be common	Genetic testing will be common	No, yes
Impact: testing mandatory to be hired	Genetic testing will be necessary to get hired	No, yes
Impact: testing for insurance premiums	Sequencing asked prior premium establishment	No, yes
Impact: genetic passport	Everyone will have a genetic passport	No, yes
Impact: segregation good/bad	There will be a segregation between “good" and “bad" genomes	No, yes
Impact: discrimination of disabled	Disabled individuals will be discriminated	No, yes
Impact: government not able to protect	Government will not be able to protect individuals	No, yes
Impact: genetic testing for infants	all infants will have their genome sequenced	No, yes
Impact: genetic testing for fœtuses	All fœtuses will undergo genetic testing	No, yes
Usage of health-related apps	Usage of health-related apps	No, yes
Usage of health-related apps for prevention	Usage of health-related apps for prevention	No, yes

#### 2.2.3. Socioeconomic factors

This set starts with a question asking the survey respondent to indicate the gender with two choices of response, male or female. For the age, we collected integers which were gathered in four classes according to our selection criteria, each class containing 25% of the sample. The classes are 25–34, 35–44, 45–54, and 55–65 years. The last selective variable is the region of residency, which can be German- or French-speaking depending on the postal code indicated by the individual. We also collect information about the respondent's nationality (Swiss/other) and education (below or above high school level). Another question concerned the professional situation, to which the responses were merged into “full-time employed,” “part-time employed,” or “other” categories. We subsequently asked about the subjective wealth of the individual which could be answered by below or above average and the marital status, which can be either “married/in a registered partnership” or “other”. Finally, the last two questions of this set dealt with health. In one question, the respondents had to rate their health from “very bad,” “bad,” “fairly good,” “good,” and “very good,” which response we classified into “bad” for the two worst levels, “average” for the middle level, and “good” for the two best levels chosen. The last question is whether the participant has a history of cancer, cardiac, or hereditary disease in the immediate family.

#### 2.2.4. Insurance factors

This set relates to the health insurance subscribed by the individual. In Switzerland, the mandatory health insurance policy has two features: the plan and the annual deductible. Hence, the first question inquires about the insurance plan, which can be of several nature: basic, Health Maintenance Organization, family doctor, or CallMed. The second question regards the deductible which can be CHF 300, CHF 500, CHF 1,000, CHF 1,500, CHF 2,000, or CHF 2,500. Usually, it is the two extremes that are favored, hence we merged the levels in the middle (CHF 500–2,000) to obtain a three-level scale. Alongside the mandatory health insurance, the individual can take out an optional complementary insurance; we, therefore, ask if he/she holds such a policy. Finally, we have a variable (insurer's app) indicating whether the person has an app from his/her health insurance for recording activity or counting steps.

#### 2.2.5. Lifestyle factors

Three questions start by inquiring the individual about his/her habits. These questions concern alcohol, cigarettes, and greens (vegetables and fruits) consumption, to which the possible responses were daily, several times a week, once a week, once every 2 weeks, once a month, less regularly, or never. We subsequently merged the responses to obtain a three-level categorical variable with “everyday,” “sometimes,” and “never” as outcomes. Physical exercise (sport) was also taken into account by a question asking the frequency at which the individual exercises. The possible answers being several times a week, once a week, less regularly, and never were pooled together to create a binary variable: at least once a week or less. To conclude this set, we dig deeper into the person's behavior by asking for his/her interest in planning for the future, together with readiness to take risks. The answers were based on an 11-point Likert scale ranging from “not interested at all” to “very interested” (future planning risk-loving).

#### 2.2.6. Political beliefs factors

Our fourth and shortest set includes three questions about political beliefs. In the first question, individuals had to express their interest in politics from the possible “not at all interested,” “slightly interested,” “fairly interested,” or “very interested” answers. The second question asked the individual to rate his/her political orientation on an 11-point Likert scale going from “left” to “right.” For the last question, we presented several political parties (with the “another several parties,” “I do not want to disclose,” and “I do not relate to any” options) and asked the person to select which party they feel the closest to. We then extracted a binary outcome indicating if the participant felt close to a political party or not.

#### 2.2.7. Sentiment factors

This last set is the largest one with 22 variables stemming from three questions and two additional health-related apps questions. In the three first questions, several statements are given to which the respondent had to chose a level of agreement on a 5-point Likert scale ranging from “strongly disagree” to “completely agree.” Subsequently, we code the answers as “is an incentive” for individuals ticking the “completely agree” and the following level and “not an incentive” for the other responses.

The first question suggests incentives to undergo genetic testing: I am curious about my genetic makeup; my results could help me take better care of my health; my results could help my relatives to take better care of their health; it could incentivize my relatives to undergo genetic testing for themselves and my results could provide useful information about my hereditary diseases or my risk cancer.

Similarly, the second question cites potential barriers to genetic testing. These hurdles being: I fear a possible discrimination; I fear the test would be too expensive; some members of my family could disapprove me taking a test; knowing my cancer risk may force me to lead a different lifestyle; I don't want to know what potential illness I might have in future; and I think my results could have a strong impact on my family's finances.

Ultimately, to capture the outlook of the individual on GT and his/her beliefs regarding GT developments, we have a series of 11 questions. The following sentences were displayed to which the respondent had to chose a level of agreement. It will be more difficult for my family members to get an insurance policy. Knowledge related to genetics will lead to fewer illnesses and longer life expectancy. It will be very common to perform GTs. Future employees will have to undergo genetic testing before being hired. Insurance companies will request a sequencing of our genome to establish premium levels. In the future, we will all have a genetic passport. There will be a segregation in our society between “good” and “bad” genomes. People with disabilities will be less accepted in society. The government will not be able to protect citizens from the negative aspects of GTs. The genome of all infants will be sequenced to establish their genetic profile and prevent development of certain diseases. Finally, all pregnant women will undergo genetic testing to determine if the fetus carries a disease.

### 2.3. Descriptive statistics

#### 2.3.1. Response variables: would you carry out such a genetic test?

**Figure 2:**
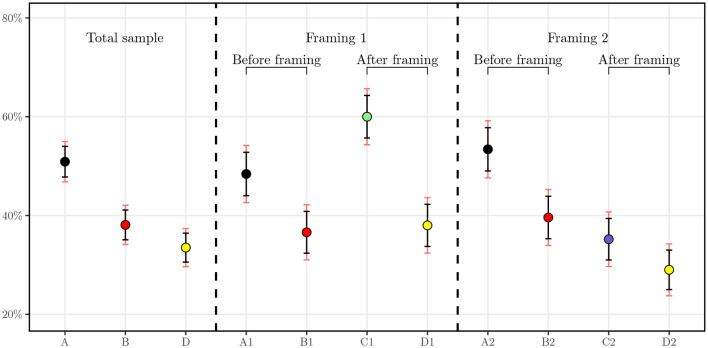
Average level of agreement with 95 and 99% confidence intervals. *A*_1_ and *A*_2_ correspond to the level of agreement for question A in each subsample of *N* = 500, respectively. *B*_1_ and *B*_2_ correspond to the level of agreement for question B in each subsample of *N* = 500. *C*_1_ and *C*_2_ correspond to the level of agreement for question C in each subsample of *N* = 500.

In this section, we perform a statistical analysis on the responses derived from the core questions presented in Section 2.2.1. In Figure 2, we display the mean values and confidence intervals for the answers to each question. The figure is divided in to three sections. The left section represents the means of the whole sample of 1,000 individuals, the middle section represents the means of the subsample presented with the first framing, insurer as a payer, and the right section, the subsample from the second framing, individual self-payer. On the left of each section of the figure, the black dot illustrates the mean level of agreement in question A in Figure 1 for the whole sample, for the insurer payer framing in the middle, and for the self payer framing on the right. The same logic applies to the red and yellow dots, which represent the means for questions B and D. In the second section, the green dot concerns the answers for those who had the insurer framing and the blue dot represents the answers for the self-payer framing. In addition, the red line represents a 99% confidence interval and the black line a 95%. The numbers corresponding to the 95% confidence interval can be found in Table 2.

**Table 2:** Average level of agreement with 95% confidence intervals.

**Question**		**Total**		**Framing 1**		**Framing 2**	
		**Agree**	**CI**_95%_	**Agree**	**CI**_95%_	**Agree**	**CI**_95%_
A	GTW	50.9%	[47.8;54.0]	48.4%	[44.0;52.8]	53.4%	[49.0;57.8]
B	GTW price bracket	38.1%	[35.1;41.1]	36.6%	[32.4;40.8]	39.6%	[35.3;43.9]
C1	GTW insurer payer			60.0%	[55.7;64.3]		
C2	GTW self payer					35.2%	[31.0;39.4]
D	DSW	33.5%	[30.6;36.4]	38.0%	[33.7;42.3]	29.0%	[25.0;33.0]
*N*		1, 000		500		500	

The abbreviations “GTW” and “DSW” stand for genetic testing willingness and data sharing willingness, respectively.

As one can first see, for the result of the baseline GTW, half of individuals (50.9%) agreed that they would be willing to undergo a GT. The distribution of this answer is not statistically different in the framed groups. Comparing with similar studies, in a randomly selected sample of 383 individuals by Smith and Croyle (32), 47.3% of the interviewees stated that they are very interested in taking a GT for colon cancer and 16.1% stated that they are not interested. More recently, in a study conducted in Saudi Arabia, authors assessed willingness to undergo presymptomatic genetic testing for Alzheimer's disease and obtained a level of agreement of 59.9% for either one of the two presented GTs and 45.1% for both tests (31). Our results hence corroborate findings for similar surveys in the literature.

Once price information is displayed, we observe the number of agreeing respondents drop from 509 individuals to 381. We note for this question that, aside from the price range of CHF 100 to CHF 400, no payer was specified. This drop can be explained by the fact that the price may be higher than expected or renders the test more tangible as knowing the price brings the individual closer to the concept of buying the product. Another explanation could be the price itself, which can be a burden for some individuals. It will be interesting to test this hypothesis in the regressions with the income variable. In addition, the two samples used in the framings do not have statistically different means with 99% confidence.

Subsequently, when the framings are applied, a clear cut appears. For the group framed with the insurer as a payer, the share of surveyed individuals agreeing to undergo the testing increases to attain 60.0% whereas those framed to be the sole payer of the test decreases as low as 35.2%. These means are statistically significantly different at 95% as their confidence intervals do not overlap. One can hypothesize that the insurer as a payer triggers more individuals to undergo the test because of the cost relief. We later test this hypothesis with regressions to understand the difference between potential drivers of the GTW.

Finally, interesting results can already be seen for the DSW of anonymized data from GTs with the health insurer. When merged together, the whole sample exhibits a DSW of 33.5%. However, in the subsamples, we observe a clear cleavage. Indeed, the two groups display statistically significantly different means at a confidence level of 95%, hinting that the role of the payer is essential in this regard. Regression analysis allows us to study this relationship and suggest possible correlation between the payer and the readiness of an individual to share health-related data with the insurer.

#### 2.3.2. Explanatory variables

In a preliminary analysis, we have a look at the descriptive statistics for the socioeconomic, insurance, lifestyle, and political beliefs in Table 3 and for the sentiment factors in Table 4. For each variable, the sample column displays the frequency of the variable in the whole sample. In the four following columns, we display the level of agreement to the questions introduced in Section 2.2.1, i.e., A–genetic testing, B–genetic testing after price display, C1–genetic testing with insurer as a payer, C2–genetic testing with the individual as a payer, and D–data sharing with the health insurer, by variable.

**Table 3:** Descriptive statistics: Willingness to undergo a genetic test and share the data per variable.

**Variable**	**Sample (%)**	**Level of agreement**					**Variable**		**Level of agreement**				
		**A**	**B**	**C1**	**C2**	**D**		**Sample (%)**	**A**	**B**	**C1**	**C2**	**D**
**Socioeconomic factors**													
**Gender**							**Health**						
Male	50.0	0.50	0.38	0.60	0.37	0.37	Bad	7.5	0.49	0.37	0.50	0.30	0.31
Female	50.0	0.52	0.38	0.60	0.34	0.30	Average	33.7	0.51	0.36	0.66	0.33	0.38
**Age**							Good	58.8	0.51	0.40	0.58	0.37	0.31
25–34	25.0	0.54	0.40	0.68	0.42	0.35	**Professional situation**						
35–44	25.0	0.58	0.42	0.71	0.37	0.37	Full time employed	51.6	0.52	0.41	0.63	0.39	0.35
45–54	25.0	0.51	0.38	0.54	0.33	0.36	Part-time employed	27.8	0.45	0.33	0.52	0.31	0.31
55–65	25.0	0.40	0.32	0.46	0.30	0.26	Other	20.6	0.58	0.37	0.64	0.32	0.33
**Nationality**							**Subjective wealth**						
Other	25.5	0.64	0.50	0.76	0.45	0.39	Below average	58.9	0.49	0.36	0.60	0.29	0.35
Swiss	74.5	0.47	0.34	0.55	0.31	0.32	Above average	41.1	0.53	0.40	0.60	0.45	0.31
**Higher education**							**Marital status**						
No	61.3	0.48	0.35	0.59	0.33	0.39	Married / Partnership	52.2	0.53	0.40	0.58	0.38	0.31
Yes	38.7	0.56	0.42	0.62	0.38	0.31	Other	47.8	0.49	0.36	0.62	0.32	0.36
**Cancer history**							**Region**						
No	54.0	0.48	0.37	0.61	0.36	0.34	French-speaking	33.0	0.55	0.38	0.67	0.35	0.29
Yes	46.0	0.55	0.39	0.60	0.34	0.33	German-speaking	67.0	0.49	0.38	0.57	0.36	0.36
**Insurance factors**													
**Insurance plan**							**Deductible**						
Basic	26.2	0.53	0.43	0.60	0.40	0.31	CHF 300	40.5	0.52	0.37	0.59	0.30	0.32
HMO	9.9	0.47	0.34	0.60	0.27	0.40	CHF {500;2000}	28.0	0.50	0.38	0.69	0.41	0.38
Family Doctor	51.9	0.50	0.36	0.60	0.35	0.35	CHF 2500	31.5	0.50	0.41	0.61	0.40	0.32
CallMed	12.0	0.51	0.38	0.62	0.32	0.25	
**Complementary insurance**							**Insurer's app**						
No	33.2	0.50	0.31	0.62	0.28	0.37	No	80.3	0.48	0.34	0.62	0.33	0.29
Yes	66.8	0.51	0.41	0.59	0.39	0.32	Yes	19.7	0.63	0.53	0.59	0.45	0.54
**Lifestyle factors**													
**Alcohol**							**Smoking**						
Everyday	3.6	0.47	0.42	0.65	0.13	0.36	Everyday	25.2	0.53	0.40	0.58	0.35	0.32
Sometimes	78.2	0.51	0.39	0.60	0.37	0.34	Sometimes	18.1	0.57	0.40	0.36	0.66	0.41
Never	18.2	0.51	0.35	0.59	0.33	0.31	Never	56.7	0.48	0.37	0.60	0.35	0.32
**Five servings of greens**							**Sport**						
Everyday	23.7	0.53	0.44	0.62	0.40	0.35	At least once a week	66.5	0.53	0.40	0.60	0.32	0.36
Sometimes	75.5	0.50	0.37	0.60	0.34	0.33	Less	33.5	0.46	0.35	0.60	0.37	0.30
Never	0.8	0.25	0.00	0.00	0.25	0.38							
**Planing for the future**							**Risk loving**						
1	3.5	0.29	0.20	0.42	0.13	0.17	1	5.3	0.45	0.36	0.54	0.37	0.26
2	1.9	0.32	0.21	0.50	0.15	0.53	2	4.5	0.36	0.22	0.52	0.08	0.22
3	3.7	0.30	0.24	0.39	0.29	0.16	3	8.5	0.41	0.27	0.53	0.21	0.31
4	4.0	0.40	0.15	0.67	0.20	0.45	4	8.7	0.54	0.43	0.61	0.34	0.36
5	6.7	0.39	0.16	0.38	0.21	0.34	5	12.7	0.42	0.31	0.48	0.29	0.28
6	11.0	0.38	0.29	0.45	0.29	0.27	6	17.4	0.54	0.40	0.60	0.46	0.32
7	17.1	0.44	0.35	0.53	0.53	0.30	7	19.0	0.52	0.36	0.61	0.37	0.34
8	22.9	0.58	0.42	0.66	0.66	0.34	8	12.4	0.57	0.49	0.67	0.40	0.40
9	13.1	0.57	0.48	0.68	0.68	0.40	9	4.7	0.64	0.43	0.67	0.35	0.38
10	16.1	0.71	0.57	0.83	0.83	0.43	10	6.8	0.60	0.51	0.76	0.43	0.47
**Political factors**													
**Interest in politics**							**Feeling close to a political party**						
No	53.2	0.49	0.36	0.60	0.34	0.33	No	44.7	0.50	0.36	0.60	0.36	0.32
Yes	46.8	0.53	0.41	0.60	0.37	0.34	Yes	55.3	0.52	0.40	0.60	0.35	0.35
**Political orientation**													
Left	4.0	0.55	0.40	0.62	0.37	0.38							
	3.8	0.53	0.42	0.60	0.43	0.24							
	8.7	0.40	0.29	0.43	0.32	0.25							
	8.1	0.60	0.58	0.61	0.45	0.37							
	9.5	0.49	0.37	0.61	0.40	0.35							
Center	30.6	0.51	0.38	0.63	0.33	0.33							
	9.1	0.52	0.36	0.57	0.37	0.43							
	10.1	0.46	0.30	0.52	0.31	0.31							
	6.3	0.44	0.35	0.51	0.33	0.25							
	3.5	0.69	0.43	0.71	0.44	0.54							
Right	6.3	0.59	0.43	0.64	0.29	0.33							
**N**	1, 000	1, 000	1, 000	500	500	1, 000		1, 000	1, 000	1, 000	500	500	1, 000

**Table 4:** Descriptive statistics: Willingness to undergo a genetic test and share the data per variable.

**Variable**	**Sample (%)**	**Level of agreement**					**Variable**		**Level of agreement**				
		**A**	**B**	**C1**	**C2**	**D**		**Sample (%)**	**A**	**B**	**C1**	**C2**	**D**
**Sentiment factors**													
**Is an incentive to undergo genetic testing**							**Is a reason not to undergo genetic testing**						
Curiosity							Impact on family finances						
No	48.2	0.26	0.17	0.16	0.40	0.25	No	72.1	0.50	0.38	0.56	0.36	0.43
Yes	51.8	0.74	0.57	0.80	0.52	0.41	Yes	27.9	0.54	0.39	0.71	0.34	0.41
Take better care of my health							Family disapproves						
No	44.5	0.25	0.18	0.37	0.17	0.23	No	79.7	0.51	0.37	0.60	0.36	0.31
Yes	55.5	0.72	0.54	0.79	0.49	0.42	Yes	20.3	0.50	0.41	0.59	0.34	0.41
Diseases risk information							Fear test too costly						
No	37.0	0.22	0.17	0.33	0.18	0.26	No	44.5	0.49	0.41	0.53	0.39	0.33
Yes	63.0	0.68	0.51	0.76	0.45	0.38	Yes	55.5	0.52	0.36	0.66	0.32	0.34
Help my relatives take better care of themselves							Do not want the info						
No	53.9	0.33	0.23	0.41	0.23	0.25	No	62.0	0.60	0.44	0.71	0.39	0.34
Yes	46.1	0.72	0.55	0.81	0.50	0.43	Yes	38.0	0.36	0.28	0.45	0.27	0.32
Could incentivize my relatives to undergo a test							Induced lifestyle changes						
No	61.7	0.36	0.26	0.45	0.24	0.26	No	56.0	0.46	0.33	0.53	0.34	0.29
Yes	38.3	0.75	0.58	0.85	0.53	0.46	Yes	44.0	0.57	0.45	0.68	0.37	0.39
**Impact of genetic tests on society**							Fear of discrimination						
More difficult family members to be insured							No	75.9	0.54	0.38	0.61	0.36	0.34
No	63.4	0.52	0.38	0.62	0.34	0.35	Yes	24.1	0.42	0.37	0.55	0.32	0.33
Yes	36.6	0.49	0.37	0.57	0.37	0.32	**Impact of genetic tests on society**						
Genetic tests will be common							Fewer illnesses and longer life expectancy						
No	62.4	0.41	0.27	0.54	0.23	0.27	No	56.0	0.39	0.26	0.47	0.24	0.26
Yes	37.6	0.68	0.56	0.71	0.56	0.44	Yes	44.0	0.66	0.54	0.76	0.50	0.43
Sequencing prior to premium establishment							Genetic testing to be hired						
No	57.4	0.52	0.40	0.59	0.35	0.35	No	81.7	0.50	0.36	0.59	0.33	0.30
Yes	42.6	0.50	0.36	0.61	0.36	0.32	Yes	18.3	0.55	0.46	0.65	0.45	0.49
Segregation between good and bad genomes							Genetic passport for everyone						
No	52.3	0.51	0.36	0.60	0.33	0.35	No	63.5	0.43	0.28	0.53	0.25	0.28
Yes	47.7	0.51	0.40	0.60	0.38	0.32	Yes	36.5	0.65	0.56	0.70	0.56	0.43
Government will not be able to protect							Discrimination toward disabled individuals						
No	49.9	0.59	0.42	0.66	0.38	0.36	No	60.4	0.57	0.39	0.63	0.37	0.36
Yes	50.1	0.43	0.34	0.55	0.31	0.31	Yes	39.6	0.44	0.36	0.55	0.33	0.30
All fœtuses will undergo genetic testing							All infants will have their genome sequenced						
No	54.9	0.45	0.32	0.49	0.30	0.30	No	60.5	0.43	0.31	0.54	0.28	0.28
Yes	45.1	0.58	0.46	0.73	0.43	0.38	Yes	39.5	0.63	0.49	0.70	0.48	0.42
**Usage of health-related apps**							**Usage of health-related apps for health prevention**						
No	29.8	0.28	0.21	0.38	0.23	0.22	No	52.3	0.53	0.37	0.64	0.31	0.38
Yes	70.2	0.61	0.45	0.70	0.40	0.38	Yes	47.7	0.70	0.55	0.76	0.50	0.39
**N**	1, 000	1, 000	1, 000	500	500	1, 000		1, 000	1, 000	500	500	1, 000	

Considering the descriptive statistics, we get a hint on possible correlations between the explanatory variables and the core questions. In the first set of variables, the socioeconomic factors, two variables stand out—age and nationality. Noticeable changes in the share of individuals who are willing to either undergo the test or share the data take place for the older group in the sample. For instance, the GTW in the group 55–65 years drops by as much as 18 percentage points (pp) when compared to the 35–44 in question A. This gap increases to 25 pp for the GTW when the insurer is the payer (C1). This difference is also true for the DSW with a disparity of 11 pp between the two groups. The older individuals in our sample seem to be reluctant to taking a GT as well as sharing the related anonymized data with their health insurer. We hence expect this effect to emerge in the regressions. The same conclusion can be drawn for the Swiss nationals in our sample. As a matter of fact, disregarding the price display or the payer of the test, they present a lower level of GTW and DSW, suggesting that Swiss are less open to these ideas. Moving on to insurance factors, some sparse but clear effects can be seen from having a complementary health insurance. The strongest positive effect for those who declared holding a complementary insurance policy intervene when cost comes into play, i.e., when the price is displayed or when the individual is the payer of the GT. On these GTW, the increase is by roughly 10 pp. In addition, the correlation between having an insurer's app for step or exercise count and GTW as well as DSW is quite strong and positive. Going to the next set, the lifestyle factors, only one variable has a clear and consistent pattern along its categories. Indeed, as the level of interest of planning for future increases, so does the share of individuals who present a positive GTW and DSW. As an example, for question A, the proportion of individuals who would be willing to get a GT rises from 29% among individuals who indicated having the lowest level of interest in future planning to 71% for those who have the highest.

For the last set on Table 3, the political factors, it is difficult to establish any hypothesis on the impact of these variables. The GTW proportions do not seem to follow a clear pattern and to display any correlation.

Table 4 contains the sentiment factors. The related variables come from three categories of questions: potential incentives for GTs, potential barriers to genetic testing, and impact of genetic testing on society. We first focus on the potential incentives to undergo genetic testing. According to our statistics, for each variable, there is a strong discrepancy between individuals who agreed with the statement and those who did not. As an example, individuals who agreed being curious about their genetic makeup is a good incentive for them to get GT are almost three times more likely to undergo genetic testing as well as share the related anonymized data with their health insurer than those who were not curious. This observation holds for all the variables in the set for a minimum difference of two-fold. Regarding the barriers, the divergences in the answers is less striking. Only not wanting to know the risks and the fear of possibly induced life changes are potential factors diminishing GTW. Finally, for variables indicating the general outlook of individuals on the GT in society, several factors show relevance. One can spot four variables: testing will be common, all fetuses as well as infants will undergo GTs, everybody will have a genetic passport, and knowledge based on genetics will increase life expectancy and promote better health. The individuals who agreed with these statements are more likely to undergo GTs. Interestingly, those who agreed that GTs will be mandatory to be hired are 63% more likely to share anonymized data with their health insurer.

### 2.4. Methodology

We perform all regressions in Section 3 using the R software. Equation (1) describes the regression of each of the interest variables, A, B, C1, C2, and D, that we denote *W*_*i*_. Each *W*_*i*_ is regressed on the five groups of factors, i.e., socioeconomic, lifestyle, insurance, political, and sentiment factors variables that form the set of variables *X*. For *W*_*i*_, we merged the possible responses into a binary variable taking the value 1 if “likely” or “very likely” was selected, and 0 otherwise. Using Akaike's information criterion (AIC), we selected the logit link function for the regression as it displayed a lower AIC. The following Equation 1 is used for all sets of explanatory variables defined by the vector *X*


(1) $$\begin{array}{l} \begin{array}{cc} g\left(W_{i}\right) & =\beta_{0j}+\underset{j,}{\sum }\beta_{Xjk}X_{jk}, \end{array} \end{array}$$

Where *j* represents each group of explanatory factors in the set *X* and *k*, each variable within this group. The β_0_ and β_*Xjk*_ coefficients correspond to the baseline, respectively, and the regression coefficients are linked to the variables *X*_*jk*_.

Furthermore, to facilitate interpretation and comparison between effects, for binary variables, we translated the β_*Xjk*_ coefficients into their probabilities (expressed in %) of obtaining 1 for *W*_*i*_. The formula for the effect of a coefficient *jk* for a particular *WIL*_*i*_ is the following:


(2) $$\begin{array}{l} \begin{array}{cc} p_{jk} & =\left(\frac{e^{\beta_{0j}+\beta_{Xjk}X_{jk}}}{1+e^{\beta_{0j}+\beta_{Xjk}X_{jk}}}-\frac{e^{\beta_{0j}}}{1+e^{\beta_{0j}}}\right)\cdot 100. \end{array} \end{array}$$

After regressing the four *W*_*i*_ variables separately on the five groups of factors, we perform an overall regression combining all factors in a single regression model. Subsequently, we select the most relevant variables using a forward and backward variable selection with the stepAIC^2^ function in R (35). This procedure allows to check for coefficients robustness and capture the most relevant explanatory variables. Finally, as an additional information, we use the randomForest package in R^3^ (36) to obtain an importance ranking of the effect of the variables on the GTW and DSW.

## 3. Results: Regression analysis and robustness checks

### 3.1. Results from regression analysis

In this subsection, we present regression results separately for each of the five sets of socioeconomic, insurance, lifestyle, political beliefs, and sentiment factors. For each of the four variables, we display the β coefficients, their equivalent *p* in terms of probabilities, and the significance. For categorical variables, the baseline is defined by the most frequent category in the sample. Following these regression results, we will present a confusion matrix and perform several robustness checks in Section 3.2.

#### 3.1.1. Socioeconomic factors

From Table 5, we observe that only few factors are significant drivers for either GTW or DSW. As expected from the literature review and the statistical analysis, except for nationality and age, the gender, education, professional status, marital status, wealth, and region of residency do not explain responses from individuals. Two regression results however confirm findings from the statistical outlook. The age and Swiss nationality do influence the GTW. As noted by the 18 pp decrease in the individuals aged 55–65 years, their GTW is distinctively lower than for other categories. They display a reduction by 17% in willingness compared to the baseline categories of 35–44 years. This decrease is however solely significant in the questions with the baseline willingness (A) and when the insurer is the payer (C1), where we observe a decrease (of −18.5%). The same observation holds for the DSW. According to our results, respondents between the ages of 55 and 65 years are 11.9% less likely to share their anonymized GTs result with the health insurer, everything else kept constant. The second variable with significant impact on questions A, B, C1, and C2 is nationality. Individuals with Swiss nationality seem less open to the idea of genetic testing, disregarding the price display or the payer, with strong significance. To conclude with this set of variables, cancer history and health present rather intriguing results. One would hypothesize that an individual who has a case of cancer in his/her close family is more enthusiastic regarding genetic testing but this hypothesis is only statistically verified for the baseline GTW, before any price is given. This inconclusive result can also be found in literature where authors either find a positive (25, 26) or a mitigated effect (27). Similarly, another belief could be that the health of the respondent comes into the decision process to undergo a GT. Our results seem to annihilate such a relationship as the variable does not present significant coefficients. Nevertheless, it is interesting to notice that when the level of agreement for genetic testing drops from question A to question B when the price range is displayed, wealthier individuals do not seem to be less affected as wealth is not show significant.

**Table 5:** Regression results for socioeconomic factors.

**Model**	**A– Baseline GTW**			**B–Price display**			**C1–Insurer payer**			**C2–Self payer**			**D–Data sharing**		
	β_*k*_	*p*_*k*_	**sig**.	β_*k*_	*p*_*k*_	**sig**.	β_*k*_	*p*_*k*_	**sig**.	β_*k*_	*p*_*k*_	**sig**.	β_*k*_	*p*_*k*_	**sig**.
**Gender (baseline: Male)**															
Female	0.099	+2.41%		0.130	+3.22%		0.172	+2.219%		0.000	+0.00%		−0.310	−7.271%	^*^
**Age (baseline: 35 – 44 years)**															
25 – 34 years	−0.113	−2.81%		−0.041	−1.00%		−0.164	−2.44%		0.278	+6.73%		−0.047	−1.13%	
45 – 54 years	−0.337	−8.39%		−0.208	−5.06%		−0.790	−13.95%	^**^	−0.129	−2.99%		−0.047	−1.124%	
55 – 64 years	−0.696	−17.19%	^***^	−0.313	−7.54%		−0.998	−18.52%	^***^	−0.196	−4.51%		−0.522	−11.87%	^**^
**Swiss nationality (baseline: No)**															
Yes	−0.630	−15.61%	^***^	−0.635	−14.66%	^***^	−0.925	−16.88%	^***^	−0.589	−12.63%	^**^	−0.292	−6.87%	
**Higher education (baseline: No)**															
Yes	0.249	+6.00%		0.164	+4.07%		−0.007	−0.13%		−0.023	−0.54%		−0.208	−4.94%	
**Professional status (baseline: Full-time employed)**															
Part-time	−0.245	−6.08%		−0.336	−8.07%		−0.609	−10.24%	^*^	−0.219	−5.02%		−0.101	−2.42%	
Other	0.322	+7.70%		−0.110	−2.69%		0.141	+1.83%		−0.206	−4.74%		0.017	+0.40%	
**Subjective wealth (baseline: Below average)**															
Above average	0.155	+3.78%		0.202	+5.03%		0.052	+0.69%		0.602	+14.81%	^**^	−0.229	−5.42%	
**Married (baseline: No)**															
Yes	0.182	+4.40%		0.139	+3.46%		−0.089	−1.30%		0.233	+5.63%		0.229	+5.64%	
**Cancer history (baseline: No)**															
Yes	0.382	+9.05%	^**^	0.178	+4.43%		0.002	−0.00%		0.061	+1.44%		−0.045	−1.08%	
**Health (baseline: Bad)**															
Average	0.069	+1.70%		0.031	+0.76%		0.662	+7.28%		−0.021	−0.50%		0.265	+6.54%	
Good	0.120	+2.93%		0.237	+5.89%		0.346	+4.24%		0.046	+1.09%		−0.005	−0.12%	
**Region (baseline: French)**															
German	−0.194	−4.81%		0.008	+0.19%		−0.351	−5.49%		0.006	+0.14%		0.313	+7.75%	^*^
Constant	0.257			−0.227			1.612		^***^	−0.488			−0.342	
*N*	1,000			1,000			500			500			1,000		

The significance levels are ^*^p < 0.05, ^**^p < 0.01, and ^***^p < 0.001. The abbreviation “GTW” stands for genetic testing willingness.

#### 3.1.2. Lifestyle factors

Among the lifestyle factors displayed in Table 6, only one variable displays a significant and consistent effect throughout all regressions: being keen on planning for the future. This variable is considered on a scale from 0 to 1, on which the individuals had to place their tendency of planning for the future. According to our results, the higher the level, the more likely is the respondent to undergo a GT, disregarding added information about the price or the payer. The same result is valid for propensity to share anonymized data. This correlation is coherent considering that in our survey, we deal with genetic testing for preventive purposes, hence for planning future medical examinations and potential diseases. Other health-related covariates do not affect individuals' decision-making, suggesting that this decision does not necessarily stem from health considerations, as already outlined by the absent correlation with the health variable in Table 5.

**Table 6:** Regression results for lifestyle factors.

**Model**	**A– Baseline GTW**			**B–Price display**			**C1–Insurer payer**			**C2–Self payer**			**D–Data sharing**		
	β_*k*_	*p*_*k*_	**sig**.	β_*k*_	*p*_*k*_	**sig**.	β_*k*_	*p*_*k*_	**sig**.	β_*k*_	*p*_*k*_	**sig**.	β_*k*_	*p*_*k*_	**sig**.
**Alcohol consumption (baseline: Everyday)**															
Sometimes	−0.185	−0.67%		0.056	+0.00%		−0.200	+0.00%		0.150	+0.75%		0.015	+0.11%	
Never	−0.299	−1.02%		0.179	+0.00%		0.020	+0.00%		−1.123	−3.38%		0.153	+1.79%	
**Cigarettes consumption (baseline: Everyday)**															
Sometimes	0.591	+3.01%	^**^	0.298	+0.00%		0.482	+0.00%		0.188	+0.96%		0.502	+6.79%	^**^
Never	0.349	+1.58%	^*^	0.223	+0.00%		−0.024	+0.00%		0.152	+0.76%		0.069	+0.75%	
**Fruits and vegetables consumption (baseline: Everyday)**															
Sometimes	1.097	+7.14%		14.932	+5.91%		16.322	+21.79%		0.298	+1.62%		−0.328	−3.36%	
Never	1.063	+6.81%		15.109	+6.97%		16.376	+22.73%		0.482	+2.87%		−0.339	−3.46%	
**Sport at least once a week (baseline: No)**															
Yes	0.253	+1.09%		0.115	+0.00%		−0.188	+0.00%		0.153	+0.77%		0.240	+2.94%	
**Level of planning for the future**															
	0.225		^***^	0.241		^***^	0.222		^***^	0.197		^***^	0.129		^***^
**Level of loving taking risks**															
	0.051			0.053			0.068			0.054			0.059		
Constant	−3.167		^***^	−17.736			−17.640			−2.967		^**^	−1.915		^**^
*N*	1,000			1,000			500			500			1,000		

The significance levels are ^*^p < 0.05, ^**^p < 0.01, and ^***^p < 0.001. The abbreviation “GTW” stands for genetic testing willingness.

#### 3.1.3. Political belief factors

Regarding political factors, the results from Table 7 are clear, there is no correlation between political belongings and GTs decisions. A plausible explanation could be that the subject is too new to be politicized. No party in Switzerland yet has formulated a clear opinion on the subject, neither on the related data. Hence, the belonging to a party or a movement of thought does not translate in a clear differentiation between individuals' responses.

**Table 7:** Regression results for political belief factors.

**Model**	**A– Baseline GTW**			**B–Price display**			**C1–Insurer payer**			**C2–Self payer**			**D–Data sharing**		
	β_*k*_	*p*_*k*_	**sig**.	β_*k*_	*p*_*k*_	**sig**.	β_*k*_	*p*_*k*_	**sig**.	β_*k*_	*p*_*k*_	**sig**.	β_*k*_	*p*_*k*_	**sig**.
**Political interest (baseline: No)**															
Yes	0.151	+3.77%		0.188	+4.47%		0.018	+0.46%		0.184	+4.44%		−0.059	−1.22%	
**Feeling close to a political party (baseline: No)**															
Yes	0.000	+0.02%		0.107	+2.50%		−0.099	−2.45%		−0.075	−1.76%		0.167	+3.58%	
**Political orientation (baseline: Left)**															
	0.191	+4.78%		−0.183	−4.15%		0.390	+9.26%		−0.338	−7.59%		0.259	+5.63%	
Constant	−0.131			−0.542		^**^	0.259			−0.486		^*^	−0.884		^***^
*N*	1,000			1,000			500			500			1,000		

The significance levels are ^*^p < 0.05, ^**^p < 0.01, and ^***^p < 0.001. The abbreviation “GTW” stands for genetic testing willingness.

#### 3.1.4. Insurance factors

In the set of insurance, we find that the chosen features of the mandatory health insurance (insurance plan and deductible) do not allow to consistently distinguish individuals who are more willing to take a GT. Three other variables, however, allow to do so. In our sample, we document that individuals who hold a complementary health insurance policy display a different behavior. More precisely, the factor comes into play when the decision to undergo a GT is faced with the cost, that is, in questions B and C2. For both cases, individuals who do own such a policy are more willing to undergo a GT by 9.33 and 14.70%, respectively, as reported in Table 8. A potential explanation could be that individuals with a complementary health insurance are less cost-conscious as the healthcare costs are alleviated. This usually leads to an increase in healthcare consumption as highlighted by Schmitz (37), thus encompassing genetic testing. Another interesting effect induced by this variable is the decrease in the DSW with the health insurer (question D): having a complementary health insurance renders individuals less likely by 7.92% to share the data. This may be correlated with the fact that the calculation of premiums for complementary health insurance in Switzerland, contrary to basic health insurance, is based, among other characteristics, on the health condition and family history. The next variable is binary, indicating whether the individual has an app from the insurer for step counting or recording exercise, participants who ticked “yes” have an increased GTW, except when the insurer is the payer, in which case, the coefficient is not significant. This outcome is rather intriguing and an underlying rationale could be that individuals who are interested in their health in the first place are more likely to download the health app. This interest then makes them more likely to be interested in performing a GT, unless when it is the insurer who is the payer, where more respondents are more interested in general, thus annihilating the significance of the difference. When it comes to sharing the anonymized data from the GT with the health insurer, the same rationale can be applied. In fact, these individuals that already share data from the app with the health insurer are 27.9% (*p* < 0.001) more willing to share GT data.

**Table 8:** Regression results for insurance factors.

**Model**	**A– Baseline GTW**			**B–Price display**			**C1–Insurer payer**			**C2–Self payer**			**D–Data sharing**		
	β_*k*_	*p*_*k*_	**sig**.	β_*k*_	*p*_*k*_	**sig**.	β_*k*_	*p*_*k*_	**sig**.	β_*k*_	*p*_*k*_	**sig**.	β_*k*_	*p*_*k*_	**sig**.
**Insurance plan (baseline: Family doctor)**															
Standard	0.164	+0.54%		0.457	+0.61%	^**^	0.016	+0.50%		0.412	+0.60%		−0.229	−5.11%	
HMO	−0.171	−4.22%		−0.113	−2.18%		−0.060	−1.45%		−0.413	+6.79%		0.040	+0.90%	
CallMed	−0.044	−1.10%		−0.034	−0.72%		0.088	+2.10%		−0.282	+7.82%		−0.584	−12.12%	^*^
**Insurance deductible (baseline: CHF 300)**															
CHF 500 – 2000	−0.134	−3.32%		−0.034	−0.72%		0.033	+0.78%		0.279	+12.53%		0.196	+4.59%	
CHF 2500	−0.024	−0.61%		0.239	+4.79%		0.085	+2.01%		0.461	+14.07%	^*^	0.053	+1.19%	
**Complementary insurance (baseline: No)**															
Yes	0.008	+0.18%		0.445	+9.33%	^**^	−0.179	−4.36%		0.535	+14.69%	^*^	−0.391	−8.46%	^**^
**Insurer's app (baseline: No)**															
Yes	0.671	+16.47%	^***^	0.793	+17.63%	^***^	0.418	+9.46%		0.530	+14.65%	^*^	1.147	+27.88%	^***^
Constant	−0.074			−1.126		^***^	0.396		^*^	−1.334		^***^	−0.581		^***^
*N*	1,000			1,000			500			500			1,000		

The significance levels are ^*^p < 0.05, ^**^p < 0.01, and ^***^p < 0.001. The abbreviation “GTW” stands for genetic testing willingness.

#### 3.1.5. Sentiment factors

Finally, our last set of variables included in the regression model in Table 9 exhibits the most significant correlations. Thereby, several variables are worth particular attention. The two first are curiosity and disease risk information. Whereas, the logic behind genetic testing take up, i.e., curiosity driving the GTW, is sound, the result generated by the second (not significant) variable is intriguing. Indeed, our model suggests that it is the simple curiosity rather than any health-related considerations, captured by the disease risk information variable, that drive the GT decision. This observation has already been made several times through our analysis with the health and lifestyle variables, thus giving further confirmation. Moreover, the curiosity is self-based as it is only enough for GT itself and does not extend to the DSW with the health insurer. Another pair of factors, however, present a pattern and they both display altruistic features. For the individuals stating that helping relatives or incentivize them to do a GT is a rather strong incentive for them to undergo one, they present different behaviors in certain cases. When the price is not yet displayed, in question A, or when it is the insurer who is the payer, in question C1, these incentives seem to differentiate respondents' choices. The effects range from 5.5% of increase in the GTW in question A for helping relatives take better care of their health to 22.4% for incentivizing a relative to undergo a test when the insurer pays for it. However, this altruism stops when individuals have to pay themselves. Ultimately, for those who could undergo a GT to incentivize relatives to do so, they are more likely to be willing to share these results with the health insurer.

**Table 9:** Regression results for sentiment factors.

**Model**	**A– Baseline GTW**			**B–Price display**			**C1–Insurer payer**			**C2–Self payer**			**D–Data sharing**		
	β_*k*_	*p*_*k*_	**sig**.	β_*k*_	*p*_*k*_	**sig**.	β_*k*_	*p*_*k*_	**sig**.	β_*k*_	*p*_*k*_	**sig**.	β_*k*_	*p*_*k*_	**sig**.
**Is an incentive to undergo genetic testing (baseline: No)**															
Curiosity	1.130	+18.92%	^***^	1.121	+15.77%	^***^	0.923	+19.38%	^***^	1.136	+19.14%	^***^	0.197	+3.15%	
Better health prevention	0.732	+10.88%	^***^	0.441	+4.83%	^*^	0.503	+9.72%		0.465	+6.35%		0.408	+6.90%	
Disease risk information	0.356	+4.66%		0.229	+2.30%		0.072	+1.28%		−0.097	−1.12%		−0.438	−5.63%	
Help relatives health prevention	0.413	+5.51%	^*^	0.340	+3.58%		0.672	+13.47%	^*^	0.288	+3.70%		0.228	+3.67%	
Incentivize relatives to undergo test	0.547	+7.65%	^**^	0.201	+1.99%		1.046	+22.37%	^**^	0.316	+4.09%		0.405	+6.85%	^*^
**Is not an incentive to undergo genetic testing (baseline: No)**															
Impact on family finances	−0.027	−0.34%		−0.210	−1.82%		0.629	+12.49%	^*^	−0.221	−2.40%		0.262	+4.25%	
Family would disapprove	0.129	+1.54%		0.239	+2.41%		−0.386	−5.79%		−0.268	−2.86%		0.273	+4.44%	
Fear test too costly	−0.059	−0.69%		−0.607	−4.46%	^***^	0.580	+11.41%	^*^	−0.593	−5.58%	^*^	−0.042	−0.60%	
Do not want to know	−0.837	−7.13%	^***^	−0.585	−4.33%	^**^	−1.343	−14.85%	^***^	−0.175	−1.94%		−0.073	−1.04%	
Induced lifestyle changes	0.236	+2.95%		0.234	+2.34%		0.560	+10.97%	^*^	−0.305	−3.20%		0.183	+2.91%	
Fear of discrimination	−0.365	−3.72%		0.198	+1.95%		0.005	+0.12%		0.116	+1.38%		−0.127	−1.79%	
**Impact of genetic tests (baseline: No)**															
Family discriminated for insurance	0.034	+0.38%		−0.028	−0.29%		−0.470	−6.87%		0.225	+2.82%		−0.259	−3.52%	
Fewer illnesses, longer life	0.110	+1.30%		0.383	+4.10%	^*^	0.567	+11.11%	^*^	0.266	+3.39%		0.331	+5.49%	^*^
Testing will be common	0.712	+10.52%	^***^	0.675	+8.09%	^***^	0.179	+3.23%		1.052	+17.34%	^***^	0.315	+5.20%	
Testing mandatory to be hired	0.229	+2.85%		0.339	+3.56%		0.105	+1.87%		0.537	+7.54%		0.932	+17.96%	^***^
Testing for insurance premiums	−0.309	−3.22%		−0.655	−4.72%	^**^	−0.026	−0.41%		−0.328	−3.42%		−0.357	−4.72%	^*^
Genetic passport for all	0.098	+1.14%		0.657	+7.82%	^***^	−0.223	−3.51%		0.592	+8.46%	^*^	0.257	+4.17%	
Segregation bad/good genomes	0.172	+2.09%		0.174	+1.70%		−0.205	−3.23%		−0.129	−1.46%		−0.342	−4.54%	
Discrimination of handicaped	−0.257	−2.73%		0.094	+0.87%		−0.270	−4.18%		−0.014	−0.20%		−0.246	−3.36%	
Government not able to protect	−0.702	−6.28%	^***^	−0.238	−2.40%		−0.600	−8.43%	^*^	−0.255	−2.74%		−0.070	−1.00%	
Sequencing of infants genome	0.247	+3.11%		−0.087	−0.81%		−0.100	−1.61%		0.152	+1.84%		0.220	+ 3.54%	
Sequencing of fœtuses genome	−0.275	−2.90%		−0.172	−1.52%		0.786	+16.10%	^**^	−0.281	−2.98%		−0.148	−2.08%	
**Usage of health-related apps (baseline: No)**															
Yes	0.784	+11.86%	^***^	0.509	+ 5.73%	^*^	0.644	+12.84%	^*^	0.021	+0.22%		0.620	+11.09%	^**^
**Usage of health-related apps for prevention (baseline: No)**															
Yes	0.166	+2.02%		0.322	+3.36%		0.087	+1.54%		0.318	+4.13%		−0.348	−4.60%	^*^
Constant	−1.868		^***^	−2.157		^***^	−1.292		^***^	−1.858		^***^	−1.501		^***^
*N*	1,000			1,000			500			500			1,000		

The significance levels are ^*^p < 0.05, ^**^p < 0.01, and ^***^p < 0.001. The abbreviation “GTW” stands for genetic testing willingness.

Regarding deterrents, cost is an issue. The fear that the test is too costly especially arises when the price is displayed in question B. With strong significance, individuals for whom the cost may be a hurdle are 4.5% less likely to undergo the test in general and 5.6% when they are the sole payer. On the contrary, when it is the insurer who is supposed to pay for the test, respondents who had an issue with the expenditure are now 11.4% more likely to undergo the test once that burden is taken away. The last significant variable in this group of barriers is the lack of desire to know what potential disease one could have in the future. Not wanting to know correlates with a decrease in 7% in the overall willingness (question A) and of 14% when the insurer bears the cost. This correlation, nevertheless disappears in the last two regressions, question C1 and DSW. Interestingly, fear of discrimination is not significant in our model, despite being fairly present in the literature [(12, 34) to name a few].

Subsequently, we capture the outlook of the respondents on genetic testing and its future. We first note that those who agree or completely agree that GT will be common are distinguishable when price comes in question from those who did not. Their belief pushes them to perform the test when the price is displayed, giving an edge compared to those who do not believe so. Another belief—that the government will not be able to protect its citizens against negative aspects of genetic testing—has a significant impact. It translates into a decrease in the GTW in models A and C1. Especially when the cost of the test is taken care of by the health insurer, the individuals who share this opinion are 8.4% less likely to undergo the test. Compelling enough, this attitude does not give a significant difference when it comes to deciding whether to share the data with the health insurer. Regarding that last question, the DSW with the health insurer, respondents who agree that testing will be mandatory before being hired are 17.9% more likely to do so. Curiously, this perspective, though, does not make them more likely to perform the test. Finally, we study the usage of health-related applications. First, the respondents who use health-related apps for step counting, sleep cycle, or women's health, for instance, have a higher propensity of accepting to undergo the test, except when they are the payers. This could be easily explained by the fact that these individuals are already familiar with health technologies and are willing to use them to monitor their health. However, these results hint again that this behavior is not driven by health considerations but rather by curiosity. This observation being backed up several times in our study is once again confirmed by the non-significance of the last usage of health-related apps for prevention factor. Regarding DSW, these last two variables present conflicting results, suggesting that those who use health apps are more likely to share the anonymized data but using this app for prevention renders them less likely to do so.

#### 3.1.6. Effect of the payer framing

In this section, we document the effect of the health insurer as a payer framing on GTW as well as DSW, as outlined in Section 2.2 and Figure 1. We capture this effect by introducing an “insurer framing” dummy variable in the GTW regressions of question C and the DSW of question D. To this aim, we first aggregate the data of questions C1 and C2, and we subsequently control for the framing by regressing the outcomes on the health insurer as a payer binary variable. By doing so, we witness a difference in outcome between the two groups, as suggested by the statistical analysis. The results of our regression in Table 10 corroborate with the observation made in the descriptive statistics analysis—the two framings present significantly different outcomes on the GTW. As the coefficient suggests, individuals who were told that it is the health insurer who should finance these GTs are 24.8% more likely to undergo the test, compared to individuals who would bear the cost of the test themselves. One can hypothesize that the insurer as a payer triggers more individuals to undergo the test because of the cost relief. To verify this conjecture, we run a subsidiary regression with the interaction term *Health insurer framing* × *Fear that test would be too costly*. When crossed with the health insurer as a payer variable, the fear of the test to be too costly is statistically significant at a 90% confidence level and has a coefficient of 0.724, thus validating the hypothesis that the health insurer as a payer alleviates the fear that the test may be too costly.

**Table 10:** Regression results for payer framing.

**Model**	**C–GTW**			**D–Data sharing**		
	β_*k*_	*p*_*k*_	**sig**.	β_*k*_	*p*_*k*_	**sig**.
**Insurer framing (baseline: No)**						
Yes	1.016	+24.81%	^***^	0.406	+9.18%	^**^
Constant	−0.585		^***^	−0.846		^***^
Observations	1,000					

The significance levels are ^**^p < 0.01, and ^***^p < 0.001. The abbreviation “GTW” stands for genetic testing willingness.

When moving to the DSW, we as well witness a difference in outcome between both groups, as suggested by the statistical analysis. Our regression coefficient provides empirical evidence that the individuals for whom the health insurer is the payer of the GT would be 9.18% more likely to share the test's anonymized data with the health insurer, when compared to individuals who are the sole payers of the GTs with 99% confidence. Making use of framings to carry another dimension into the analysis of GTW and DSW, we highlight the critical importance of the payer of these tests. From the findings in this framework emerge a new perspective in which the health insurer and the insured establish a collaboration relationship. When the health insurer pays for the GT to be undergone by the insured, which can lead to actionable information, this can be viewed as an investment into the individual's health capital. In return, the insured shares the anonymized data. A possible explanation of this significantly different behavior could be that it stems from a latent feeling of indebtedness toward the health insurer, rather than collaboration. However, it is not possible to determine the extent to which this might play a role in practice.

### 3.2. Robustness checks and additional analysis

We now assess the robustness of our results by performing several checks. First, in Table 11, we produce confusion matrices on 10,000 bootstrapped samples, providing the mean accuracy for the five models in regard to each variable of interest. The mean accuracy spreads between 51 and 79%. The best performing model at explaining GTW is sentiment-related. Its accuracy ranges between 71 and 79%. Unsurprisingly, the model that performs the worst concerns political belief factors: there are no significant variables for this model. Finally, it is usually the DSW that is best explained (model D).

**Table 11:** Prediction accuracy per regression model.

**Model**	**A–Baseline GTW**	**B–Price display**	**C1–Insurer payer**	**C2–Self payer**	**D–Data sharing**
Socioeconomic	0.61	0.63	0.65	0.66	0.64
Insurance	0.55	0.63	0.60	0.65	0.69
Lifestyle	0.62	0.65	0.64	0.66	0.67
Political belief	0.51	0.62	0.60	0.65	0.67
Sentiment	0.79	0.76	0.79	0.76	0.71

The abbreviation “GTW” stands for genetic testing willingness.

#### 3.2.1. Total regression, StepAIC, and reduced form regression results

For the second robustness check, we calibrate several regression models to test the sensitivity of our coefficients. In a robust model, coefficients should almost not vary when new variables are introduced, a case that we simulate by running a regression making use of all our variables. Another aim of conducting a regression comprising all the variables is to subsequently reduce the model with a selection based on the AIC. This procedure keeps the variables that improve the explanatory power of the model and hence provide another mapping of variable importance in GT decision. In Table 12, we display both the total regression model and the reduced model. A first observation, we can make regards for the robustness of the coefficients. Expectedly, we can notice that coefficients of significant variables vary much less than those that are not significant. For instance, health, a variable that is not significant, has a coefficient that changes from 0.120 to −0.153 in the case of good health reported by the surveyed person. The factor curiosity, on the contrary, has a stable coefficient with only a minor change from 1.130 to 1.150 from the reduced to the total regression. Another interesting perspective is the change in the significance of the coefficients. Merging all the variables together has confirmed previous findings pointing at the importance of sentiment-related factors. Indeed, in the total regression, other sets of variables which displayed a few significant drivers in the separate models lose their importance when merged together, leaving almost solely significance to the sentiment factors. Finally, if we take a closer look at the analysis of the DSW, we notice that two variables remain highly significant and bear a strong coefficient: having a health insurer's application and the insurer's framing (framing 2). The latter even displays a stronger coefficient, increasing from 0.409 to 0.575 while remaining significant at a 99% level of confidence. These findings confirm the importance of the relationship between the insurer and the respondent in the DSW.

**Table 12:** Regression results for the overall and reduced models.

**Model**	**A–Baseline GTW**				**B–Price display**				**C1–Insurer payer**				**C2–Self payer**				**D–Data sharing**			
	**Total**		**Reduced**		**Total**		**Reduced**		**Total**		**Reduced**		**Total**		**Reduced**		**Total**		**Reduced**	
**Gender (baseline: Male)**																				
Female	0.088				0.193				0.407				−0.179				−0.264		−0.324	*
**Age (baseline: 35 – 44 years)**																				
25 – 34 years	0.021		0.041		0.081				0.369		0.297		0.496				0.032			
45 – 54 years	−0.224		−0.154		−0.178				−0.880	*	−0.877	*	0.216				0.010			
55 – 64 years	−0.626	*	−0.567	*	−0.194				−0.510		
**Swiss nationality (baseline: No)**																				
Yes	−0.458	*	−0.487	*	−0.529	**	−0.555	**	−0.836	*	−0.782	*	−0.541		−0.509	*	−0.085			
**Higher education (baseline: No)**																				
Yes	0.231				0.040				0.028				−0.317				−0.254		−0.229	
**Professional status (baseline: Full-time employed)**																				
Part-time	0.074		0.074		−0.127				−0.421				0.097				0.032			
Other	0.581	*	0.561	*	0.091				0.174				0.126				0.075			
**Subjective wealth (baseline: Below average)**																				
Above average	0.251				0.209				0.040				0.885	**	0.657	**	−0.303		−0.250	
**Married (baseline: No)**																				
Yes	0.266		0.301		−0.077				−0.131				0.067				0.133			
**Cancer history (baseline: No)**																				
Yes	0.266				−0.039				−0.277				−0.234				−0.123			
**Health (baseline: Bad)**																				
Average	−0.038				0.214				0.755				0.332				0.294		0.458	
Good	−0.153				0.246				0.023				0.100				−0.086		0.131	
**Region (baseline: French-speaking)**																				
German-speaking	0.047				0.495	*	0.507	**	−0.202				0.339				0.558	**	0.580	***
**Alcohol consumption (baseline: Everyday)**																				
Sometimes	0.055				0.306		0.327		0.054				−0.018				0.156			
Never	0.041				1.026	*	0.934	*	0.563				−0.199				0.461			
**Cigarettes consumption (baseline: Everyday)**																				
Sometimes	0.655	**	0.627	**	0.063				0.169				−0.143				0.284			
Never	0.448	*	0.451	*	0.198				−0.660				0.301				−0.016			
**Fruits and vegetables consumption (baseline: Everyday)**																				
Sometimes	0.338				15.473		14.815		15.171				0.322				−0.740			
Never	0.247				15.603		14.971		15.142				0.622				−0.651			
**Sport at least once a week (baseline: No)**																				
Yes	0.215				−0.207				−0.400				−0.071				0.107			
**Level of planning for the future**																				
	0.023				0.060				0.090				−0.011				0.041			
**Level of loving taking risks**																				
	0.020				−0.005				0.121		0.095		−0.031				0.026			
**Insurance plan (baseline: Family doctor)**																				
Standard	0.160				0.495	*	0.407	*	0.443				0.605				−0.310		−0.278	
HMO	−0.350				−0.273		−0.261		−0.149				−0.099				0.095		0.128	
CallMed	−0.254				−0.327		−0.299		−0.381				−0.367				−0.735	**	−0.760	**
**Insurance deductible (baseline: CHF 300)**																				
CHF 500 – 2000	−0.203				−0.047				0.072				0.469		0.519		0.190			
CHF 2500	−0.084				0.274				0.022				0.780	*	0.566	*	0.134			
**Complementary insurance (baseline: No)**																				
Yes	0.002				0.681	***	0.680	***	−0.131				0.613	*	0.419		−0.423	*	−0.447	**
**Insurer's app (baseline: No)**																				
Yes	0.402		0.340		0.674	**	0.565	**	0.127				0.200				0.930	***	1.043	***
**Insurer's framing (baseline: No)**																				
Yes																	0.575	***	0.549	***
**Is an incentive to undergo genetic testing (baseline: No)**																				
Curiosity	1.115	***	1.126	***	1.104	***	1.200	***	0.951	**	0.843	**	1.084	***	1.211	***	0.154			
Better health prevention	0.733	**	0.848	***	0.392		0.553	**	0.418		0.575		0.680		0.501		0.505	*	0.419	*
Disease risk information	0.407		0.473	*	0.264				0.316				−0.022				−0.365			
Help relatives health prevention	0.410		0.418	*	0.350		0.496	**	0.556		0.686	*	0.438		0.423		0.096			
Incentivize relatives to undergo test	0.537	*	0.516	*	0.263				1.312	***	1.175	***	0.373				0.486	*	0.561	**
**Is a reason not to undergo genetic testing (baseline: No)**																				
Impact on family finances	−0.031				−0.203				0.779	*	0.691	*	−0.340				0.297		0.335	*
Family would disapprove	0.071				0.289				−0.553		−0.515		−0.280				0.200			
Test too costly	0.015				−0.594	**	−0.589	***	0.615	*	0.583	*	−0.631	*	−0.740	**	−0.030			
Do not want to	−0.854	***	−0.754	***	−0.685	***	−0.614	**	−1.364	***	−1.429	***	−0.250				−0.138			
Induced lifestyle changes	0.183				0.149				0.356		0.502		−0.432				0.142			
Fear of discrimination	−0.400		−0.429	*	0.309		0.350		0.136				0.303				−0.039			
**Impact of genetic tests on society (baseline: No)**																				
Family discriminated for insurance	0.027				0.110				−0.749	*	−0.626	*	0.373				−0.224			
Fewer illnesses, longer life	0.058				0.311		0.348	*	0.715	*	0.707	**	0.055				0.401	*	0.439	**
Testing will be common	0.719	***	0.807	***	0.752	***	0.800	***	0.264				1.142	***	0.918	***	0.422	*	0.514	**
Testing mandatory to be hired	0.138				0.344				0.083				0.707		0.474		0.830	***	0.861	***
Testing for insurance premiums	−0.367		−0.312		−0.838	***	−0.699	***	−0.098				−0.435				−0.437	*	−0.460	*
Genetic passport for all	0.107				0.744	***	0.680	***	−0.353				0.837	**	0.656	*	0.183			
Segregation bad/good genomes	0.195				0.268				0.027				−0.106				−0.367		−0.358	*
Discrimination of handicaped	−0.255				0.010				−0.231				−0.137				−0.323		−0.347	
Government not able to protect	−0.723	***	−0.661	***	−0.266				−0.393		−0.611	*	−0.382		−0.468		0.009			
Sequencing of infants genome	0.262		0.307		−0.102				−0.130				0.262				0.214			
Sequencing of fœtuses genome	−0.427	*	−0.377		−0.280				0.729	*	0.658	*	−0.500				−0.162			
**Usage of health-related apps (baseline: No)**																				
Yes	0.737	**	0.929	***	0.348		0.403		0.624		0.796	**	−0.092				0.510	*	0.431	*
**Usage of health-related apps for prevention (baseline: No)**																				
Yes	0.273				0.409	*	0.385	*	0.316				0.407		0.368		−0.211			
**Political interest (baseline: No)**																				
Yes	0.044				−0.026				0.132				−0.085				−0.039			
**Belong to a political party (baseline: CHF 300)**																				
Yes	−0.249				0.007				−0.791	*	−0.662	*	−0.297				−0.062			
**Political orientation (baseline: left)**																				
	0.176				−0.431				1.740	**	1.413	*	−0.820		−0.827		0.223			
Constant	−2.784	*	−1.981	***	−18.859		−17.768		−17.287		−1.449	*	−2.725		−2.206	***	−1.759		−2.040	***
*N*	1,000				1,000				500				500				1,000			

The significance levels are ^*^p < 0.05, ^**^p < 0.01, and ^***^p < 0.001. The abbreviation “GTW” stands for genetic testing willingness.

Using the same Table 12 but now looking at the coefficients of the reduced model, we have another evidence of the importance of sentiment variables as well as insurance-related ones. The variables remaining in the reduced models mostly come from the sentiment factors and for the DSW decision variables from the insurance factors.

#### 3.2.2. Random forest

As a last analysis and robustness check for the importance of factors in the decision process of GTW or data sharing, we report results obtained from the random forest application.^4^ In our case, we classify each respondent whether he/she is likely to undergo a GT, respectively, to share the data or not. The algorithm performs the best classification and we extract the ranking of each variable, which is presented in Table 13 (see column “RF”). The variables considered as the most important are the ones that allow as soon as possible to classify the highest number of individuals into either group with the highest accuracy. We find that the first ranks stem from the incentive sentiment factors for GTW, for insurer's app usage and genetic testing impact for DSW.

**Table 13:** Regression results for the reduced model and comparison with the variable ranking from random forest modeling.

**Model**	**A–Baseline GTW**		**B–Price display**		**C1–Insurer payer**		**C2–Self payer**		**D–Data sharing**	
	**Reduced**	**RF**	**Reduced**	**RF**	**Reduced**	**RF**	**Reduced**	**RF**	**Reduced**	**RF**
**Gender (baseline: Male)**										
Female									✓	
**Age (baseline: 35 – 44 years)**										
25 – 34 years	✓				✓^*^	(10)				
45 – 54 years	✓				✓^*^	(10)				
55 – 64 years	✓^*^				✓	(10)				
**Swiss nationality (baseline: No)**										
Yes	✓^*^		✓^**^		✓^*^		✓^*^			
**Higher education (baseline: No)**										
Yes									✓	
**Professional status (baseline: Full-time employed)**										
Part-time	✓									
Other	✓^*^									
**Subjective wealth (baseline: Below average)**										
Above average							✓^**^	(10)	✓	
**Married (baseline: No)**										
Yes	✓									
**Cancer history (baseline: No)**										
Yes										
**Health (baseline: Bad)**										
Average									✓	
Good									✓	
**Region (baseline: French-speaking)**										
German-speaking			✓^**^						✓^***^	
**Alcohol consumption (baseline: Everyday)**										
Sometimes			✓							
Never			✓^**^							
**Cigarettes consumption (baseline: Everyday)**										
Sometimes	✓^**^									
Never	✓^*^									
**Fruits and vegetables consumption (baseline: Everyday)**										
Sometimes			✓							
Never			✓							
**Sport at least once a week (baseline: No)**										
Yes									
**Level of planning for the future**										
				(10)						
**Level of loving taking risks**										
					✓					
**Insurance plan (baseline: Family doctor)**										
Standard			✓^*^						✓	
HMO			✓						✓	
CallMed			✓						✓^**^	
**Insurance deductible (baseline: CHF 300)**										
CHF 500 – 2000							✓			
CHF 2500							✓^*^			
**Complementary insurance (baseline: No)**										
Yes			✓^***^				✓		✓^**^	
**Insurer's app (baseline: No)**										
Yes	✓		✓^**^				✓		✓^***^	(1)
**Insurer's framing (baseline: No)**										
Yes									✓^***^	
**Is an incentive to undergo genetic testing (baseline: No)**										
Curiosity	✓^***^	(1)	✓^***^	(1)	✓^**^	(2)	✓^***^	(1)		
Better health prevention	✓^***^	(2)	✓^**^	(2)	✓	(1)	✓	(2)	✓^*^	
Disease risk information	✓^*^	(3)		(3)		(3)		(5)		
Help relatives health prevention	✓^*^	(5)	✓^**^	(4)	✓^*^	(5)	✓	(4)		(7)
Incentivize relatives to undergo test	✓^*^	(4)		(6)	✓^***^	(4)			✓^**^	(6)
**Is not an incentive to undergo genetic testing (baseline: No)**										
Impact on family finances					✓^*^				✓^*^	
Family would disapprove					✓					
Test too costly			✓^***^		✓^*^		✓^**^	(9)		
Do not want to	✓^***^	(8)	✓^**^	(8)	✓^***^					
Induced lifestyle changes										
Fear of discrimination	✓^*^		✓							
**Impact of genetic tests (baseline: No)**										
Family discriminated for insurance					✓^*^					
Fewer illnesses, longer life		(9)		(8)	✓^*^	(7)	✓^**^	(7)	✓^**^	(3)
Testing will be common	✓^***^	(7)	✓^***^	(5)			✓^***^	(3)	✓^**^	(4)
Testing mandatory to be hired							✓		✓^***^	(2)
Testing for insurance premiums	✓		✓^***^						✓^*^	
Genetic passport for all			✓^***^	(7)			✓^*^	(8)		(9)
Segregation bad/good genomes									✓^*^	
Discrimination of handicaped									✓	
Government not able to protect	✓^***^				✓^*^		✓			
Sequencing of infants genome	✓									(5)
Sequencing of fœtuses genome	✓			(9)	✓^*^					
**Usage of health-related apps (baseline: No)**										
Yes	✓^***^	(6)	✓		✓^**^				✓^*^	
**Usage of health-related apps for prevention (baseline: No)**										
Yes		(10)	✓^*^	(9)		(6)	✓			(10)
**Political interest (baseline: No)**										
Yes										
**Belong to a political party (baseline: CHF 300)**										
Yes					✓^*^					
**Political orientation (baseline: Left)**										
					✓		✓			
*N*	1,000		1,000		500		500		1,000	

The significance levels are

* *p* < 0.05,

** *p* < 0.01,

and

*** *p* < 0.001.

The abbreviation “GTW” stands for genetic testing willingness.

## 4. Discussion and conclusion

GTs by essence give access to personalized health. Understanding what drives the decision to undergo these tests and the associated fears is crucial for personalized health-oriented policies. The two-fold aim of this article is reflected in the design of the *ad hoc* survey. To fill the gap in the literature and better understand health-related decisions, we first analyzed the factors influencing genetic testing uptake as well as the sharing of the anonymized data from the GT with the health insurer. To do so, we ran regressions on five sets of variables susceptible to influence individuals' behavior regarding their GTW decision, including socioeconomic, insurance, lifestyle, political beliefs, and sentiment factors. We find that socioeconomic (age and nationality) and lifestyle factors (smoking habits and planning for the future) have little or inconsistent significant influence, while the insurance (complementary health insurance and insurer's app usage) and sentiment factors (e.g., curiosity and health prevention) present strong and significant results regarding GTW. These findings are corroborated by random forest modeling robustness checks. For instance, the perception of GT as a mean for health prevention pushes the individual's propensity for testing by more then 10 pp. Furthermore, following a GT, an individual is 27.6% more likely to share the anonymized results with the health insurer if the individual already has an app from the insurer. Curiosity about one's genetic making is, overall, the strongest explanatory variable throughout all our models. Respondents who stated that curiosity for them would be an incentive to undergo genetic testing are on average 18% more likely to undergo the test, disregarding the display of the price or the payer.

Subsequently, making use of framings in the design of our survey, we are able to shed light on the relationship between GTW along with the related data sharing and the nature of the payer of this GT, namely the individual itself or the health insurer. Our model is able to capture the critical importance of the payer in the decision process of undergoing the test and sharing anonymized genetic data. We provide empirical evidence of the impact of the health insurer as a payer on to GTW and DSW. Precisely, when the health insurer should be the payer, GTW and DSW increase by 24.8 and 9.2%, respectively.

The empirical results that this article provides are relevant for several streams of research. On the academic side, we lay the ground for a deeper understanding of the presence of a payer on health decisions as well as sharing of health-related data. We confirm findings from the extant body of literature on the relevance of number of factors influencing the GTW (cf. Section 2). As a novel result, for insurance, practitioners, we present the relevance of collaboration between clients and their insurance. With that in mind, an interesting avenue for further research may be, for example, how the amount of the insurance coverage of genetic testing influences preferences. However, while we believe that our set of variables is quite extensive, further uncaptured idiosyncratic characteristics may play a role in the decision process. For example, our study disregards ethical aspects, the mean of delivery of GT information, clinical counseling, and the limitations of GTs. Furthermore, privacy concerns are important in the context of personalized health [see Deruelle et al. (38)]. We conducted our research on survey-collected data, which intrinsically carries several biases. Self-reported data include flaws such as social desirability [see Gittelman et al. (39)] or health specific biases [documented in Bound et al. (40)]. Hence, results are to be taken with hindsight and a robustness test on another type of data (such as panel data, to get rid of confounding variables) could improve the results. In addition, the results we obtain are valid for Switzerland or countries under the same healthcare system. Extending this research to other models of healthcare would further increase the knowledge on health decisions.

## Data availability statement

The raw data supporting the conclusions of this article will be made available by the authors, without undue reservation.

## Ethics statement

Ethical review and approval was not required for the study on human participants in accordance with the local legislation and institutional requirements.

## Author contributions

VK performed the statistical analysis and wrote the first draft of the manuscript. All authors contributed to conception and design of the study, contributed to manuscript revision, read, and approved the submitted version.

## Footnotes

^1^Despite the prospects, clinical evidence is scarce, and individuals must be aware that false positives (artifacts) and false negatives (i.e., “reassuring” results) are possible. This is why users have to be literate about the interpretation of the result before basing health and clinical decisions on it [see, e.g., Horton et al. (5)]. The mean of delivery and the consequences on the lives of the patients receiving genetic testing information represent a real challenge in the clinical application of such technology [see, e.g., Ensenauer et al. (6)]. In addition, there are still limitations to the usability of the results given the complexity of translating genetic data into individualized recommendations for, e.g., nutrition [see, e.g., Ordovas et al. (7) and Verma et al. (8)].

^2^See https://www.rdocumentation.org/packages/MASS/versions/7.3-54/topics/stepAIC.

^3^See https://cran.r-project.org/web/packages/randomForest/randomForest.pdf.

^4^A random forest is composed of a multitude of decision trees, which are used as supervised categorization algorithms. That is, for a decision tree, the data is provided to the machine which then tries to use the available variables to classify the “object” into either category.

## References

[1] Perkins BA Caskey CT Brar P Dec E Karow DS Kahn AM. Precision medicine screening using whole-genome sequencing and advanced imaging to identify disease risk in adults. Proc Natl Acad Sci USA. (2018) 115:3686. 10.1073/pnas.1706096114 29555771 PMC5889622

[2] Jin J Wu X Yin J Li M Shen J Li J. Identification of genetic mutations in cancer: challenge and opportunity in the new era of targeted therapy. Front Oncol. (2019) 9:263. 10.3389/fonc.2019.00263 31058077 PMC6477148

[3] Lima ZS Ghadamzadeh M Arashloo FT Amjad G Ebadi MR Younesi L. Recent advances of therapeutic targets based on the molecular signature in breast cancer: genetic mutations and implications for current treatment paradigms. J Hematol Oncol. (2019) 12:38. 10.1186/s13045-019-0725-6 30975222 PMC6460547

[4] Su P. Direct-to-consumer genetic testing: a comprehensive view. Yale J Biol Med. (2013) 86:359–365. 24058310 PMC3767220

[5] Horton R Crawford G Freeman L Fenwick A Wright CF Lucassen A. Direct-to-consumer genetic testing. BMJ. (2019) 367:15688. 10.1136/bmj.l5688 31619392 PMC6829432

[6] Ensenauer RE Michels VV Reinke SS. Genetic testing: practical, ethical, and counseling considerations. Mayo Clinic Proc. (2005) 80:63–73. 10.1016/S0025-6196(11)62960-1 15667031

[7] Ordovas JM Ferguson LR Tai ES Mathers JC. Personalised nutrition and health. BMJ. (2018) 361:2173. 10.1136/bmj.k2173 29898881 PMC6081996

[8] Verma M Hontecillas R Tubau-Juni N Abedi V Bassaganya-Riera J. Challenges in personalized nutrition and health. Front Nutr. (2018) 5:117. 10.3389/fnut.2018.00117 30555829 PMC6281760

[9] Horne J Madill J O'Connor C Shelley J Gilliland J. A systematic review of genetic testing and lifestyle behaviour change: are we using high-quality genetic interventions and considering behaviour change theory? Lifestyle Genomics. (2018) 11:49–63. 10.1159/000488086 29635250

[10] McGeoch L Saunders CL Griffin SJ Emery JD Walter FM Thompson DJ. Risk prediction models for colorectal cancer incorporating common genetic variants: a systematic review. Cancer Epidemiol Biomarkers Prev. (2019) 28:1580. 10.1158/1055-9965.EPI-19-0059 31292139 PMC7610631

[11] Fogel AL Jaju PD Li S Halpern-Felsher B Tang JY Sarin KY. Factors influencing and modifying the decision to pursue genetic testing for skin cancer risk. J Am Acad Dermatol. (2017) 76:829–835.e1. 10.1016/j.jaad.2016.11.050 28087134

[12] Dalpe G Feze IN Salman S Joly Y Hagan J Levesque E. Breast cancer risk estimation and personal insurance: a qualitative study presenting perspectives from canadian patients and decision makers. Front Genet. (2017) 8:128. 10.3389/fgene.2017.00128 28983318 PMC5613157

[13] Sweeny K Ghane A Legg AM Huynh HP Andrews SE. Predictors of genetic testing decisions: a systematic review and critique of the literature. J Genet Counsel. (2014) 23:263–88. 10.1007/s10897-014-9712-9 24719248

[14] Armstrong K Calzone K Stopfer J Fitzgerald G Coyne J Weber B. Factors associated with decisions about clinical BRCA1/2 testing. Cancer Epidemiol Biomarkers Prev. (2000) 9:1251–4. 11097234

[15] Kopits IM Chen C Roberts JS Uhlmann W Green RC. Willingness to pay for genetic testing for alzheimer's disease: a measure of personal utility. Genetic Testing Mol Biomarkers. (2011) 15:871–5. 10.1089/gtmb.2011.0028 21749214 PMC3241735

[16] Lerman C Marshall J Audrain J GomezCaminero A. Genetic testing for colon cancer susceptibility: anticipated reactions of patients and challenges to providers. Int J Cancer. (1996) 69:58–61. 10.1002/(SICI)1097-0215(19960220)69:1&lt;58::AID-IJC15&gt;3.0.CO;2-G 8600064

[17] Hall MA McEwen JE Barton JC Walker AP Howe EG Reiss JA. Concerns in a primary care population about genetic discrimination by insurers. Genet Med. (2005) 7:311–6. 10.1097/01.GIM.0000162874.58370.C0 15915082

[18] Allain DC Friedman S Senter L. Consumer awareness and attitudes about insurance discrimination post enactment of the genetic information nondiscrimination act. Familial Cancer. (2012) 11:637–44. 10.1007/s10689-012-9564-0 22890887

[19] Haga SB Barry WT Mills R Ginsburg GS Svetkey L Sullivan J. Public knowledge of and attitudes toward genetics and genetic testing. Genet Testing Mol Biomarkers. (2013) 17:327–35. 10.1089/gtmb.2012.0350 23406207 PMC3609633

[20] Clayton EW Halverson CM Sathe NA Malin BA. A systematic literature review of individuals' perspectives on privacy and genetic information in the United States. PLoS ONE. (2018) 13:e0204417. 10.1371/journal.pone.0204417 30379944 PMC6209148

[21] Miron-Shatz T Hanoch Y Katz BA Doniger GM Ozanne EM. Willingness to test for BRCA1/2 in high risk women: influenced by risk perception and family experience, rather than by objective or subjective numeracy? Judgment Decis Making. (2015) 10:15. 10.1017/S1930297500005180

[22] Tubeuf S Willis T Potrata B Grant H Allsop M Ahmed M. Willingness to pay for genetic testing for inherited retinal disease. Eur J Human Genet. (2015) 23:285–91. 10.1038/ejhg.2014.111 24916649 PMC4326707

[23] Wessel J Gupta J de Groot M. Factors motivating individuals to consider genetic testing for type 2 diabetes risk prediction. PLoS ONE. (2016) 11:e0147071. 10.1371/journal.pone.0147071 26789839 PMC4720283

[24] Blouin-Bougie J Amara N Bouchard K Simard J Dorval M. Disentangling the determinants of interest and willingness-to-pay for breast cancer susceptibility testing in the general population: a cross-sectional Web-based survey among women of Québec (Canada). BMJ Open. (2018) 8:e016662. 10.1136/bmjopen-2017-016662 29487071 PMC5855474

[25] Abdul Rahim HF Ismail SI Hassan A Fadl T Khaled SM Shockley B. Willingness to participate in genome testing: a survey of public attitudes from Qatar. J Hum Genet. (2020) 65:1067–73. 10.1038/s10038-020-0806-y 32724056 PMC7605429

[26] Sun S Li S Ngeow J. Factors shaping at-risk individuals' decisions to undergo genetic testing for cancer in Asia. Health Soc Care Commun. (2020) 28:1569–77. 10.1111/hsc.12981 32196825

[27] Sanderson S Wardle J Jarvis M Humphries S. Public interest in genetic testing for susceptibility to heart disease and cancer: a population-based survey in the UK. Prev Med. (2004) 39:458–64. 10.1016/j.ypmed.2004.04.051 15313084

[28] Rosenstock IM. Historical origins of the health belief model. Health Educ Monogr. (1974) 2:328–35. 10.1177/109019817400200403 299611

[29] Gollust SE Gordon ES Zayac C Griffin G Christman MF Pyeritz RE. Motivations and perceptions of early adopters of personalized genomics: perspectives from research participants. Public Health Genomics. (2012) 15:22–30. 10.1159/000327296 21654153 PMC3225236

[30] Kauffman TL Irving SA Leo MC Gilmore MJ Himes P McMullen CK. The NextGen study: patient motivation for participation in genome sequencing for carrier status. Mol Genet Genomic Med. (2017) 5:508–15. 10.1002/mgg3.306 28944234 PMC5606895

[31] Alanazy MH Alghsoon KA Alkhodairi AF Binkhonain FK Alsehli TN Altukhaim FF. Public willingness to undergo presymptomatic genetic testing for Alzheimer's disease. Neurol Res Int. (2019) 2019:2570513. 10.1155/2019/2570513 30941216 PMC6421033

[32] Smith K Croyle R. Attitudes toward genetic testing for colon-cancer risk. Am J Public Health. (1995) 85:1435–8. 10.2105/AJPH.85.10.1435 7573633 PMC1615625

[33] Bosompra K Flynn BS Ashikaga T Rairikar CJ Worden JK Solomon LJ. Likelihood of undergoing genetic testing for cancer risk: a population-based study. Prev Med. (2000) 30:155–66. 10.1006/pmed.1999.0610 10656843

[34] Cameron LD Sherman KA Marteau TM Brown PM. Impact of genetic risk information and type of disease on perceived risk, anticipated affect, and expected consequences of genetic tests. Health Psychol. (2009) 28:307–16. 10.1037/a0013947 19450036

[35] Venables WN Ripley BD. Random and mixed effects. In: Modern Applied Statistics with S. Statistics and Computing. New York, NY: Springer (2002). p. 271-300.

[36] Breiman L. Random forests. Mach Learn. (2001) 45:5–32. 10.1023/A:1010933404324

[37] Schmitz H. More health care utilization with more insurance coverage? Evidence from a latent class model with German data. Appl Econ. (2012) 44:4455–68. 10.1080/00036846.2011.591733

[38] Deruelle T Kalouguina V Trein P Wagner J. Designing privacy in personalized health: an empirical analysis. Bid Data Soc. (2023) forthcoming.

[39] Gittelman S Lange V Cook WA Frede SM Lavrakas PJ Pierce C. Accounting for social-desirability bias in survey sampling. J Advert Res. (2015) 55:242–54. 10.2501/JAR-2015-006

[40] Bound J Brown C Mathiowetz N. Chapter 59-measurement error in survey data. Handbook Econometr. (2001) 5:3705–43. 10.1016/S1573-4412(01)05012-7

