# Supplementary material for: On the determinants and the role of the payers in the uptake of genetic testing and data sharing in personalized health
Source: Front Public Health. 2023 Mar 2;11:920286. doi: 10.3389/fpubh.2023.920286 (PMC10017738; doi:10.3389/fpubh.2023.920286)
Supplement: Supplementary file 1 [file Data_Sheet_1.PDF]

Supplementary Material

SURVEY QUESTIONS

General information and selection of participants

Question A1: Gender.

What is your gender? *Answer options: male; female.*

Question A2: Age.

What is your age? *Numeric answer.*

Question A3: Region.

What is the postal code of your main residence? *Numeric answer.*

Usage, storage and sharing of data

In the following questions, we refer to two types of data collected through the different means below:

Smartphone health apps or connected devices that record data relating to your health, such as number of steps, sports activities, heart rate, weight, sleep quality or stress level.

Do-it-yourself blood or genetic tests to be sent to a laboratory to determine possible food intolerances, to suggest an optimal exercise plan or to assess the risk of certain hereditary diseases (such as cancer, for example).

Question B1: Technologies usage.

Do you or would you use the technologies below to record your data?
1. Apps or connected devices
2. Blood or genetic tests
Answer options: not likely; unlikely; likely; very likely.

Question B2+: Possible reasons of acceptance of usage of apps or connected devices usage.

(Only if answered likely; very likely *in question B1.1.)* You have indicated that you rather want to use connected applications or devices. What are the reasons? Several answers are possible. *Answer options: curiosity; surveillance; coaching sport / health; too expensive; other.*

[. . .]

Genetic Tests

Introductory paragraph

For the following questions, we focus on **genetic testing**. Some of these tests determine the risk for hereditary diseases, for instance breast cancer for women and prostate cancer for men. These tests can then be used to plan the frequency of preventive medical examinations (e.g. mammograms) or to improve lifestyle (diet, physical activity) in order to decrease or postpone the risk of disease.

Question C1: Incentives to undergo genetic testing.

Which of the following reasons might incentivize you to take a genetic test? For each reason, indicate your level of agreement.
In random order:
1. I am curious about my genetic makeup.
2. My results could help me to take better care of my health.
3. My results could help my relatives to take better care of their health.
4. It could incentivize my relatives to undergo a genetic testing for themselves.
5. My results could provide me useful information about hereditary diseases or my cancer risks.
Answer options: five levels from strongly disagree *to* totally agree.

Question C2: Reasons not to undergo genetic testing.

Which of the following reasons might incentivize you to take a genetic test? For each reason, indicate your level of agreement.
In random order:
1. I fear a possible discrimination.
2. I fear that the test would be too expensive.
3. Some members of my family could disapprove me taking a test.
4. Knowing my cancer risk may force me to lead a different lifestyle.
5. I don't want to know what potential illness I might have in the future.
6. I think that my results could have a strong impact on my family's finances.
Answer options: five levels from strongly disagree *to* totally agree.

Question C3: Genetic testing willingness.

Would you carry out such a genetic test? *Answer options: not likely; unlikely; likely; very likely.*

Additional information disclosure A genetic test costs between CHF 100 and CHF 400.

Question C4: Genetic testing willingness.

Taking these costs into account, would you carry out such a genetic test? *Answer options: not likely; unlikely; likely; very likely.*

Framing for financing of genetic tests.

Framing C Some people say that genetic testing should be paid for by health insurance.

Question C5c: Genetic testing willingness.

Would you carry out such a genetic test? *Answer options: not likely; unlikely; likely; very likely.*

Framing D Some people say that genetic testing should be paid for by the individuals themselves.

Question C5d: Genetic testing willingness.

Would you carry out such a genetic test? *Answer options: not likely; unlikely; likely; very likely.*

Question C6: Genetic testing willingness.

If you were to perform a genetic test, would you share the anonymized test data with your health insurer? *Answer options: not likely; unlikely; likely; very likely.*

Question C7: Impact of genetic testing on society.

We are now considering the impact of genetic testing on society. For each of the following reasons, indicate your level of agreement.
In random order:
1. It will be more difficult for my family members to get an insurance policy.
2. Knowledge related to genetics will lead to fewer illnesses and longer life expectancy.
3. It will be very common to perform a genetic test.
4. Future employees will have to undergo genetic testing before being employed.
5. Insurance companies will request a sequencing of our genome to establish premiums level.
6. In the future we will all have a genetic passport.
7. There will be a separation in our society between the "good" genomes and the "bad" ones.
8. People with disabilities will be less accepted in society.
9. The government will not be able to protect citizens from the negative aspects of genetic testing.
10. The genome of all infants will be sequenced to establish their genetic profile and prevent the development of certain diseases.
11. All pregnant women will undergo genetic testing to determine if the fœtus carries a disease.
Answer options: five levels from strongly disagree *to* totally agree.

Control variables

Question D1: Physical activity.

How often do you do gymnastics, fitness or sports?
Answer options: several times a week; once a week; less regularly; never.

Question D2: Lifestyle.

How often do you consume these products?
1. Alcohol.
2. Cigarettes, cigars, e-cigarette.
3. Five servings of fruits and vegetables.
Answer options: daily; several times a week; once a week; once every two weeks; once a month; less regularly; never.

Question D3: Health.

How do you rate your general health? Is it... *Answer options: very good; good; fairly good; bad; very bad.*

Question D4: Cancer history.

Do you have a history of cancer, heart disease or hereditary disease in the immediate family? *Answer options: yes; no.*

Question D5: Risk aversion and planning.

How do you evaluate yourself personally?
1. Are you generally interested in planning for the future?
2. Are you generally willing to take risks?
Answer options: ten levels from not interested at all *to* very interested.

Question D6: Health insurance.

Which health insurance model do you personally have for compulsory basic insurance?
Answer options: standard model; HMO model; family doctor; CallMed.

Question D7: Health insurance.

What is your annual deductible in the compulsory basic insurance? *Answer options: CHF 300; CHF 500; CHF 1 000; CHF 1 500; CHF 2 000; CHF 2 500.*

Question D8: Health insurance.

Do you have complementary insurance? *Answer options: yes; no.*

Question D9: Health insurance.

Do you use an app from your insurer to record the number of steps or sports activities? *Answer options: yes; no.*

Question D10: Political behaviour.

For each of the following reasons, indicate your level of agreement.
1. It is the role of the state to plan and guarantee the financing of health care for the entire population through social insurance.
2. It is the role of the state to intervene in the economy to reduce inequalities.
3. It is the role of the state to store and use data.
4. It is the role of the state to regulate the storage and sharing of data.
5. I support the sharing of my social insurance data to create a more efficient social system and with less fraud.
6. I support the sharing of my health data to help research for medical progress.
7. I support the sharing of my bank account data to optimize the fight against tax fraud.
8. I support the sharing of my telephone data (connections and movement profiles) to improve crime and terrorism prevention.

Answer options: five levels from strongly disagree *to* totally agree.

Question D11: Political behaviour.

In general, are you interested by politics?
Answer options: not at all interested; slightly interested; fairly interested; very interested.

Question D12: Political behaviour.

In politics, we talk about left and right. Where would you rank yourself? *Answer options: eleven levels from* left *to* right.

Question D13: Political behaviour.

Do you feel close to a political party and, if so, which one? *Answer options: PS; Les Verts; PDC; PLR; UDC; another / several parties; I do not want to disclose; I do not relate to any.*

Question D14: Political behaviour.

You find that the budget of the Confederation, the cantons and the municipalities in Switzerland allocated to health care is ... *Answer options: too high; sufficient; insufficient; too low.*

Question D15: Socioeconomic status.

What is your marital status? *Answer options: married / registered partnership; other.*

Question D16: Socioeconomic status.

About the composition of your household :
1. How many people live in your household including yourself?
2. How many children under the age of 18 live in your household?
Numeric answer.

Question D17: Socioeconomic status.

What is your current professional situation?
Answer options: full-time employee or self-employed (working time over 80%); employed or self-employed part-time (working time less than or equal to 80%); at home / without paid work; retired; other.

Question D18: Socioeconomic status.

What is your highest level of education?
Answer options: compulsory school; initial vocational education (vocational maturity, federal certificate of competence, federal vocational education certificate); high school diploma or general culture school (high school diploma, specialized diploma, certificate of general culture) ; higher vocational education and training (federal certificate, federal diploma, higher school diploma); university/EPF, pedagogical or specialized schools (Bachelor, Master, PhD).

Question D19: Socioeconomic status.

Taking into account your total household income and wealth, would you rather say that you are ... *Answer options: in a modest situation.; in a slightly below average situation.; in a slightly above average situation.; a well-to-do household..*

Question D20: Socioeconomic status.

What is your nationality? In case of dual nationality, please indicate your nationality at birth. *Answer options: nationality*

Question D21: Socioeconomic status.

How many years have you lived in Switzerland? *(Only if did not answer* CH *in question D20.) Answer options: < 1 year; between 1 and 5 years; between 5 and 10 years; more than 10 years.*
